# Evaluation and Error Characterization of a Multi-View 3D Markerless Motion Capture System for Human Kinematics

**DOI:** 10.3390/s26216773

**Published:** 2026-10-26

**Authors:** Ziao Chen, Francisco Espinoza, Sajeda Al-Hammouri, Christopher J. Arellano, Kavan Hazeli

**Affiliations:** 1 Department of Psychology, The University of Arizona, Tucson, AZ 85721, USA; ziaochen@arizona.edu; 2 Department of Orthopaedic Surgery, The University of Arizona, Tucson, AZ 85724-5064, USA; espinozaf@arizona.edu (F.E.); arellanoc@arizona.edu (C.J.A.); 3 Department of Biomedical Engineering, The University of Arizona, Tucson, AZ 85721-0020, USA; sajedaalhammouri@arizona.edu; 4 Biomedical Systems and Informatics Engineering, Yarmouk University, Irbid 21163, Jordan; 5 Department of Mechanical Engineering, University of California, Riverside, CA 92521, USA

**Keywords:** markerless motion capture, pose estimation, validation, MediaPipe Pose, Vicon system, multi-view triangulation, motion analysis, Functional Movement Screen, low-cost motion capture, biomechanical assessment

## Abstract

Markerless motion capture using RGB cameras offers an accessible alternative to marker-based systems, but its measurement properties require further evaluation. We evaluated a three-camera MediaPipe Pose system against a Vicon marker-based reference during deep squat and in-line lunge exercises in six healthy adults. Analyses examined calibration, landmark visibility, camera configuration, systematic joint position offsets, and residual variability. Calibration root mean square error (RMSE) was below 3 mm and small relative to joint position disagreement. For the tested arrangement, three cameras increased data availability and reduced errors relative to camera pairs when compared at time points with sufficient landmark visibility in all three views. The mean trial-level median joint position error was 45.4 mm, mean segment orientation error was 9.6°, and mean overall angle error was 6.4°. Subject-specific offset correction in marker-based segment frames reduced these values to 28.6 mm, 7.4°, and 4.6°, respectively, in the same trials used to estimate the offsets. Cross-validation yielded similar improvements within subjects and smaller improvements across subjects, while still requiring marker-based segment frames. Residual variability showed subject-dependent directional patterns within this cohort. These findings characterize agreement with the reference and underscore the need to consider visibility, landmark definitions, and participant variability when interpreting performance.

## 1. Introduction

Quantitative analysis of human movement provides objective measures of joint motion, posture, balance, and movement strategy for biomechanical research, with potential applications in clinical assessment, sports performance, injury prevention, and rehabilitation. Conventional marker-based motion capture systems provide detailed 3D kinematic measurements but generally require specialized laboratories, multiple high-speed cameras, trained personnel, substantial setup time, and reflective markers attached to participants. These requirements can limit the individuals and movements studied and make routine assessment outside the laboratory time-consuming and technically challenging [1,2]. Markerless motion capture addresses some of these limitations by estimating body landmarks directly from video. Systems based on standard RGB cameras may reduce equipment and preparation requirements, allow participants to move in normal clothing, and support data collection in more natural environments [2,3]. These characteristics have motivated applications in gait and postural assessment, evaluation of neurological and musculoskeletal conditions, rehabilitation monitoring, analysis of daily and functional movements, athletic performance assessment, and return-to-sport testing [3,4,5]. Markerless systems may also support more frequent or large-scale assessments and real-time feedback for exercise, physical therapy, and movement training. Their suitability depends on the accuracy and robustness of the estimated kinematics, making validation against established motion capture methods essential.

Recent advances in deep-learning-based pose estimation have enabled markerless systems that reconstruct 3D human motion from standard RGB cameras. Nakano et al. [6] demonstrated multi-camera reconstruction using OpenPose and validated joint positions against laboratory motion capture. MediaPipe Pose [7] has also been used in lightweight systems. Dill et al. [8] compared stereo triangulation with monocular 3D estimation, and Bajrami et al. [9] additionally evaluated multi-view fusion and two- and three-camera configurations. Ceriola et al. [10] compared pose estimators for hip and knee kinematics, while OpenCap [11] combines HRNet or OpenPose detections with anatomical-marker augmentation and biomechanical constraints.

Camera configuration and unreliable detections also affect reconstruction. Samani et al. [12] evaluated angular agreement across camera pairs during static poses and lifting, and Yin et al. [13] modeled how camera number and geometry affect measurement precision in optical metrology. Fortini et al. [14] combined confidence-based observation rejection and weighted reconstruction to address unreliable detections and occlusion. Together, these studies show that performance depends on the estimator, camera configuration, reconstruction method, and outcome definition. For lightweight systems intended for functional movement assessment, validation should also characterize how landmark visibility and systematic joint offsets affect reconstructed positions and downstream kinematics.

In this study, we evaluated a low-cost markerless motion capture system against a Vicon marker-based reference system. The system used MediaPipe Pose to detect 2D landmarks in images from three RGB cameras connected to a typical mid-range PC, then reconstructed 3D poses by triangulation. Six healthy adults performed two exercises from the Functional Movement Screen, the deep squat and in-line lunge [15], while both systems recorded simultaneously. We compared joint positions, segment orientations, and angle metrics and evaluated calibration residuals alongside joint position disagreement. We also evaluated how landmark visibility and its threshold affected accuracy and data availability, compared two- and three-camera reconstructions on identical valid observations, and assessed data availability separately. Finally, we characterized systematic joint offsets and residual variability, using cross-validation to assess the consistency of offset correction within subjects and its transferability across subjects. These analyses characterize how landmark visibility, camera configuration, and systematic offsets affect reconstructed positions and downstream kinematics in a lightweight multi-camera system.

## 2. Materials and Methods

### 2.1. Motion Capture Systems

#### 2.1.1. Markerless 3D Pose Estimation System

The markerless 3D pose estimation system consisted of three RGB cameras (Spedal 920 Pro webcams) connected via USB to a typical mid-range PC. The cameras operated at a resolution of 1024×768 and a frame rate of 30 Hz. The resolution balanced image quality and computational cost and was considered sufficient for the capture volume because MediaPipe Pose converts the human-body bounding box into a 256×256 image for pose estimation. In each acquisition loop, a frame was grabbed from each camera before the images were retrieved, and one host-computer timestamp was assigned to the three-frame set. Frames from the same loop were used together for triangulation.

**Figure 1:**
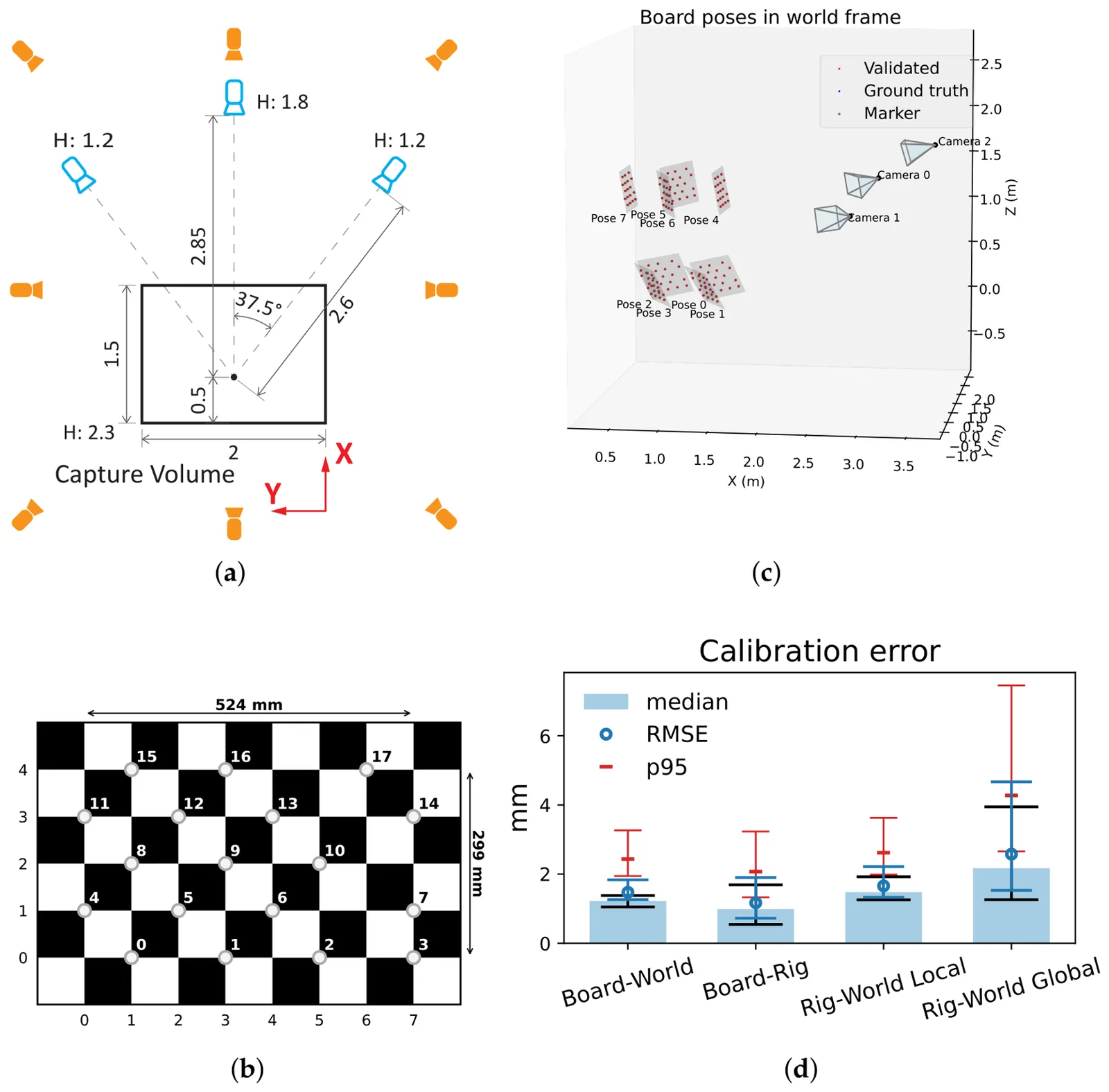
(**a**) Schematic layout of the experimental setup with the marker-based (orange cameras) and markerless (blue cameras) motion capture systems. All dimensions are in meters. H denotes height. The X- and Y-axes of the world reference frame lie in the horizontal plane. (**b**) Layout of 18 markers on a subset of corners of the checkerboard pattern for calibration evaluation. (**c**) Eight board poses in the world reference frame; ‘Ground truth’ in the legend denotes ideal corner coordinates defined by the measured board dimensions. (**d**) Error statistics for the checkerboard corner points after transformations between reference frames (‘Board’ denotes the checkerboard, ‘Rig’ the markerless system, and ‘World’ the marker-based system). RMSE denotes root mean square error. ‘Board–World’ and ‘Rig–World’ both denote local errors. Error bars represent 95% confidence intervals across sessions.

The capture volume was cuboidal (1.5×2×2.3 m; Figure 1a), with a focal point near the back edge of the capture area and sufficient height to accommodate most individuals standing with their arms raised overhead. The cameras, designated left, center, and right, were positioned in front of the capture volume, with the front direction corresponding to the X-axis, and had a horizontal angular separation of 37.5° between adjacent cameras. The outer-camera separation of 75° was selected to maintain a compact spatial footprint.

#### 2.1.2. Marker-Based Vicon System

We used a Vicon motion capture system as the marker-based reference. The system consisted of eight Vicon Vero infrared cameras that recorded at 100 Hz and were mounted on steel bars fixed to the walls. Its capture volume encompassed that of the markerless system (Figure 1a). Joint positions were calculated using Vicon’s 57-marker full-body “Plug-in Gait” model [16] and subsequent processing pipelines.

### 2.2. Data Collection

#### 2.2.1. Calibration Board and World Coordinate System

The world coordinate system was established by calibrating the marker-based system and was distinct from the coordinate system of the markerless system. Both systems were calibrated before each of the three data collection sessions.

To establish a transformation between the reference frames of the two systems, we used a calibration board and a marker set corresponding to selected corner positions on the checkerboard pattern. Markers were fixed at 18 specific corner positions on a 3D-printed frame that could be attached to the top of the calibration board. The positions were arranged asymmetrically to permit unique marker identification (Figure 1b). The ideal positions of the corresponding corners in the board reference frame were assigned according to the physically measured dimensions of the checkerboard pattern.

Eight poses of the board within the capture volume were recorded by both systems during each session (Figure 1c). The checkerboard pattern in each pose was detected by all three cameras of the markerless system, and the 3D corner positions were reconstructed in the rig reference frame. A transformation between the rig and world reference frames was then estimated using the corner and marker positions measured by the two systems.

#### 2.2.2. Participants and Exercises

Six healthy adults (three males and three females; two per session), aged 18–30 years and ranging in height from 157.4 to 180.3 cm, performed the deep squat and in-line lunge from the Functional Movement Screen [15]. Each participant completed three repetitions per set: one set of deep squats and two sets of in-line lunges, with one for each side (Figure S1). Participants faced the center camera (X direction) during the deep squat, left (Y direction) during the left lunge, and right (opposite Y direction) during the right lunge. Each set was recorded simultaneously by both systems as a single trial.

### 2.3. Data Processing

The marker-based data were collected and processed using Vicon Nexus 2.16 software and exported in CSV format. All markerless-system data acquisition, processing, and analysis steps were performed using Python 3.12.7.

#### 2.3.1. Camera Calibration

Camera parameters were calibrated using a checkerboard pattern (Figure 1b) before every session. The intrinsic parameters were first calibrated separately for each camera, and the extrinsic parameters were then estimated using bundle adjustment, which simultaneously minimized the checkerboard pattern’s reprojection error across all cameras [17].

#### 2.3.2. Pose Estimation

Pose estimation was performed independently for each camera view using MediaPipe Pose (model complexity 1) with multiprocessing. The resulting 2D landmarks were triangulated using the direct linear transformation (DLT) algorithm [18]. When a visibility threshold was applied, landmarks below the threshold were excluded; at least two views were required for reconstruction. Each reconstructed 3D landmark was assigned the minimum visibility score among the 2D landmarks used. We tracked 16 joints derived from the MediaPipe Pose landmarks (shoulder, elbow, wrist, hip, knee, ankle, heel, and foot index on both sides) and calculated two additional landmarks: the chest and pelvis, defined as the midpoints of the shoulders and hips, respectively. Each midpoint was assigned the minimum visibility score of its two constituent landmarks.

Corresponding joint positions were obtained from the marker-based system. Plug-in Gait joint locations are model-derived estimates rather than direct measurements of true anatomical joint centers. The heel and foot index are not joints in the skeletal model, so markers placed near these locations were used as substitutes. Their differences from MediaPipe landmarks may reflect physical marker placement, as well as landmark estimation. Visibility in the Vicon system was binary (i.e., present or missing).

#### 2.3.3. Spatial Coordinate Systems

In each session, corner and marker positions from different board poses were used to estimate transformations between reference frames in two steps. First, a similarity transformation between each pair of board, rig, and world frames was estimated from the n=18 corners or markers in each pose. The residual error was defined as the local error. Second, a global rig–world transformation was estimated using all corner and marker positions across the m=8 poses, accounting for the board-to-marker offset described below. The residual error was defined as the global error. A similarity transformation includes rotation, translation, and scaling, i.e., Tx=sRx+t, where x is the point being transformed, *R* is a rotation matrix, t is a translation vector, and *s* is a scaling factor. Scaling bias (Table S2) was calculated as 100(s−1)%.

##### Local Transformation

The ideal position of the *i*-th corner in the board frame is denoted by $x_{i}$. For the *i*-th corner (or marker) in the *j*-th board pose, the measured position is denoted by $y_{ij}$ for the corner in the rig frame and by $z_{ij}$ for the marker in the world frame. Similarity transformations among the three reference frames were estimated using the Umeyama method [19], which minimizes the sum of squared distances between corresponding points:

(1) $$\begin{array}{c} T_{BR}^{\left(j\right)}=\underset{T}{\mathrm{argmin}}\sum_{i=1}^{n}{∥Tx_{i}-y_{ij}∥}^{2},\\ T_{BW}^{\left(j\right)}=\underset{T}{\mathrm{argmin}}\sum_{i=1}^{n}{∥Tx_{i}-z_{ij}∥}^{2},\\ T_{RW}^{\left(j\right)}=\underset{T}{\mathrm{argmin}}\sum_{i=1}^{n}{∥Ty_{ij}-z_{ij}∥}^{2}, \end{array}$$

where $T_{BR}^{\left(j\right)}$, $T_{BW}^{\left(j\right)}$, and $T_{RW}^{\left(j\right)}$ are the similarity transformations between reference frames for the *j*-th pose, and *B*, *R*, and *W* in the subscripts denote the board, rig, and world frames, respectively. A common scaling factor constrained the transformation between each pair of reference frames across all poses, e.g., $s_{RW}^{\left(j\right)}=s_{RW}$ for all *j*.

##### Global Transformation

A single similarity transformation could not directly map the corner positions $y_{ij}$ to the marker positions $z_{ij}$ across all poses because the markers on the 3D-printed frame were offset from the board surface. As board orientation changed, this fixed offset changed direction in the world frame, so the combined point configurations were not similar even without measurement error. Parallel board planes could satisfy the similarity condition, but the offset would introduce an unknown translation into the global mapping. We therefore formulated a variant of the hand–eye calibration problem [20] that jointly estimated the global transformation and board-to-marker offset:

(2) $$\underset{T_{RW},\;T_{BM}}{\mathrm{argmin}}\sum_{j=1}^{m}\sum_{i=1}^{n}{∥T_{RW}y_{ij}-T_{MW}^{\left(j\right)}T_{BM}x_{i}∥}^{2},$$

where $T_{RW}$ is the global rig–world similarity transformation and $T_{BM}$ is the board–marker rigid transformation ($s_{BM}=1$) describing the fixed offset. The marker–world transformation $T_{MW}^{\left(j\right)}=T_{BW}^{\left(j\right)}$ is the previously estimated board–world transformation for pose *j*: both use the same ideal coordinates $x_{i}$, assigned to the markers in the marker frame and the corners in the board frame. The problem was solved using the SciPy least_squares function with a Huber loss and a scale parameter of 2 mm to reduce the influence of outliers. Board planes were intentionally nonparallel to avoid a numerically unstable, degenerate solution. The global transformation converted all markerless measurements from the rig frame to the world frame for subsequent analysis.

#### 2.3.4. Synchronization Between Systems

The timestamps in recordings from the two systems were generated independently by their respective host computers. Trials were synchronized between systems by maximizing the cross-correlation of the joint positions estimated by the two systems. Markerless-system data were first resampled to the 100 Hz sampling rate of the marker-based system using quadratic interpolation for lag estimation only. A subset of joints (elbow, wrist, knee, ankle, chest, and pelvis) was selected to calculate the cross-correlation of joint positions along each axis, and these cross-correlations were summed across the selected joints and axes. The time lag that maximized the summed cross-correlation was used to align the timestamps from the two systems. Marker-based-system data were then interpolated at the aligned native timestamps of the markerless system using quadratic interpolation. Final error statistics were evaluated at these native markerless time points; the intermediate 100-Hz grid did not provide additional independent observations.

#### 2.3.5. Joint Positions in Segment Reference Frames

Body segments were treated as nearly rigid, allowing markers on each segment to establish its reference frame. Each joint was associated with a segment, and its position was transformed into that segment’s coordinates at each time point. A set of markers served as a reference template for estimating segment pose. Each marker was assigned manually estimated coordinates along the medial–lateral, anterior–posterior, and superior–inferior anatomical axes (Table A1).

A rigid transformation from the world frame to each segment frame was estimated at each time point using the Umeyama method, analogous to the board–world transformation (Section Local Transformation). Because each subject’s marker configuration could deviate from the template, generalized Procrustes analysis (GPA) [21] was used to estimate a subject-specific mean configuration. GPA alternates rigid alignment of marker configurations across time with averaging of the aligned coordinates, minimizing squared marker-position differences after accounting for segment translation and rotation. The mean configuration is aligned with the template to retain the anatomical-axis orientation and then used to estimate segment-frame transformations (Appendix A).

Marker position data from all exercises performed by each subject were used to estimate that subject’s mean marker configuration. The rigid transformation of each segment at each time point was then estimated and applied to the joint positions measured by both systems to obtain their coordinates in the segment frame.

### 2.4. Statistical Analysis

#### 2.4.1. Joint Position Offsets and Residual Variability

Joint positions in the segment reference frame were denoted by $x_{j}$ and $y_{j}$ at time point *j* for the marker-based and markerless systems, respectively. The reference-relative joint position difference was defined as $e_{j}=y_{j}-x_{j}$, and the systematic offset was defined as the mean difference $d=E_{j}e_{j}$ across all time points and trials for each subject. The proportion of squared error explained by the offset was calculated as ${∥d∥}^{2}/E_{j}{∥e_{j}∥}^{2}$. Corrected markerless joint positions were obtained by subtracting the subject-specific offset in the segment frame.

Variability in joint offsets across subjects was evaluated using the coefficient of variation (CV) of offset magnitude and the angular deviation of offset direction in the segment reference frame. The CV is the ratio of the standard deviation to the mean. Angular deviation [22], expressed in radians, is defined as $\sqrt{2(1-R_{\mathrm{unbiased}})}$, where $R_{\mathrm{unbiased}}^{2}=\frac{n}{n-1}\left(R^{2}-\frac{1}{n}\right)$ is an unbiased estimator of the squared mean resultant length for a small sample. The mean resultant length for *n* subjects is $R=∥\frac{1}{n}\sum_{i=1}^{n}d_{i}/∥d_{i}∥∥$.

Variability in joint estimation was analyzed using principal component analysis (PCA) of joint positions $x_{j}$ and $y_{j}$ in the segment reference frame after subtracting their mean positions over time. These residuals quantify within-segment position variation and were not assumed to be purely random error. Explained variance ratios along the three principal axes indicated directional preference. Consistency across subjects was quantified using the linearity index of the first-principal-axis directions. The index uses the largest eigenvalue of an orientation tensor to measure concentration around a common axis, treating opposite directions as equivalent. A value of 1 indicates perfect parallel alignment, whereas values near 0 indicate little shared directional preference; a small-sample correction was applied (Appendix B.1).

#### 2.4.2. Performance Metrics

Using joint positions from both systems in the world frame, we calculated position error statistics, per-axis and multivariate correlations, segment orientation errors, and angle errors across time points within each trial.

Here, joint position error denotes disagreement with the marker-based reference, not independently established anatomical localization error. Its statistics included the median, 95th percentile, and root mean square error (RMSE) of the distances between positions estimated by the two systems. Correlations along each world axis were calculated using the Pearson correlation coefficient (PCC) and Lin’s concordance correlation coefficient (CCC) [23]. The PCC measures a linear relationship and is invariant to scale and bias, whereas the CCC measures agreement, including differences in scale and bias (Appendix B.2).

The remaining statistics depend on multiple joint positions, so time points missing any required joint were excluded. Multivariate correlations were calculated using the RV-coefficient [24] and matrix-based concordance correlation coefficient (MCCC) [25], multivariate generalizations of the PCC and CCC, respectively. The RV-coefficient measures global structural similarity and is invariant to rotation, translation, and scaling, whereas the MCCC measures overall agreement (Appendix B.3).

The segment orientation error was defined as the angle between the segment direction vectors measured by the two systems. The angle metrics included inter-segmental and segment angles. Inter-segmental angles were defined as the angles between adjacent body segments, whereas segment angles were defined as the angles between a segment direction vector and an anatomical axis. For example, ‘right upper arm elevation’ is the angle between the right upper arm direction and the longitudinal axis. ‘Hip rotation’ is the angle between the left-to-right hip direction and the frontal axis. ‘Torso incline’ and ‘torso balance’ are the angles between the torso direction (from pelvis to chest) and the sagittal and frontal axes, respectively.

Overall joint position statistics were evaluated by pooling data across all joints, whereas overall statistics for segment orientation and angle metric errors were calculated by averaging across segments and metrics, respectively.

The wrist and elbow joints and their associated metrics were excluded from evaluations of the in-line lunge because these joints had low visibility in both systems (Figure S5a).

#### 2.4.3. Cross-Validation of Offset Correction

Offsets estimated and evaluated using the same trials were termed in-sample corrections. We assessed their consistency and transferability using cross-validation. In three-fold within-subject cross-validation (leave-one-repetition-out), one repetition from each of a subject’s trials was held out per fold. Subject-specific offsets and mean marker configurations defining the segment reference frames were estimated from the remaining repetitions and used to correct the held-out repetitions. In six-fold across-subject cross-validation (leave-one-subject-out), a common offset was obtained by averaging the height-normalized offsets of the other five subjects, scaling it to the held-out subject’s height, and applying it to that subject’s measurements. Both corrections required marker-based segment reference frames, including those of the held-out participant in across-subject validation.

## 3. Results

### 3.1. Calibration Error and Temporal Misalignment

The markerless rig frame and marker-based world frame were established separately after calibration. To compare measurements in a common frame, we recorded eight board poses per session using 18 checkerboard corners reconstructed in the rig frame and corresponding markers measured in the world frame (Figure 1b,c; front view in Figure S2).

These measurements quantified baseline uncertainty associated with calibration, reconstruction, checkerboard detection, and marker placement, for comparison with joint position disagreement. Local error was evaluated over 18 corners per pose after Umeyama alignment (Methods Section Local Transformation). Global error was evaluated over 8×18=144 corners after hand–eye calibration accounted for the board-to-marker offset (Methods Section Global Transformation).

#### 3.1.1. Local Error

Ideal corner positions were assigned according to the checkerboard geometry in the checkerboard reference frame (board frame). The transformation between the board and world frames (board–world) was estimated for each pose using the ideal corner positions and the marker positions measured in the world frame. The local error after the board–world transformation comprised marker-placement misalignment relative to the ideal corner positions and 3D reconstruction error arising from calibration and reconstruction by the marker-based system.

The transformation between the board and rig frames (board–rig) was estimated for each pose using the ideal corner positions and the reconstructed corner positions in the rig frame. The local error after the board–rig transformation comprised deviations in checkerboard-pattern detection from the ideal corner positions and 3D reconstruction error arising from calibration and reconstruction by the markerless system.

The transformation between the rig and world frames (rig–world) was estimated for each pose using the corner positions in the rig frame and marker positions in the world frame. The local error after the rig–world transformation comprised all the error sources described above.

These error statistics quantified the baseline local uncertainty of both systems over the area of the checkerboard pattern (524×299 mm) within the capture volume. The average local error was below 2 mm for both systems (Figure 1d).

#### 3.1.2. Global Error

Global rig–world alignment accounted for the fixed offset between the board surface and the markers above it. The optimization aligned reconstructed corners transformed directly from rig to world with ideal corners transformed first from board to marker and then from marker to world. The marker–world transformation had already been estimated from marker positions for each pose; the global rig–world transformation and fixed board–marker offset were estimated jointly (Methods Section Global Transformation).

Global error statistics were calculated from the misalignment of corner positions in the world frame after application of the optimized transformations. The global error was slightly larger than the local error and showed greater variance across sessions (Figure 1d and Table S1).

The board–world, board–rig, and rig–world transformations included scaling. Their mean absolute scaling bias was less than 1% in all cases (Table S2).

Calibration RMSE was below 3 mm in all cases and was small relative to the joint position disagreement analyzed below.

#### 3.1.3. Temporal Misalignment

Joint movement between camera captures can introduce position discrepancies when cameras are imperfectly synchronized. To estimate the scale of this effect, we assumed inter-camera timing differences of up to one frame interval (33 ms at 30 FPS) and multiplied marker-based joint speeds by that interval. Estimated displacement was 2.3 mm at the median speed across all joints and trials and 13.2–24.2 mm at individual joints’ 95th-percentile speeds (Table S3). These values estimate displacement rather than reconstruction error; actual timing differences were not independently measured.

Separately, because alignment between the markerless and marker-based systems was estimated from joint trajectories, we checked how sensitive the reported errors were to the chosen time lag. Comparing linear and quadratic lag estimation and testing shifts of plus or minus one native markerless frame changed the uncorrected cohort means by less than 1 mm for position error and 0.2° for segment orientation and overall angle errors. These averages were therefore relatively stable under the tested local alignment changes; the procedures and full results are provided in Supplementary Section S2 (Figure S15).

### 3.2. Effects of Landmark Visibility and Camera Configuration

#### 3.2.1. Landmark Visibility and Joint Position Error

**Figure 2:**
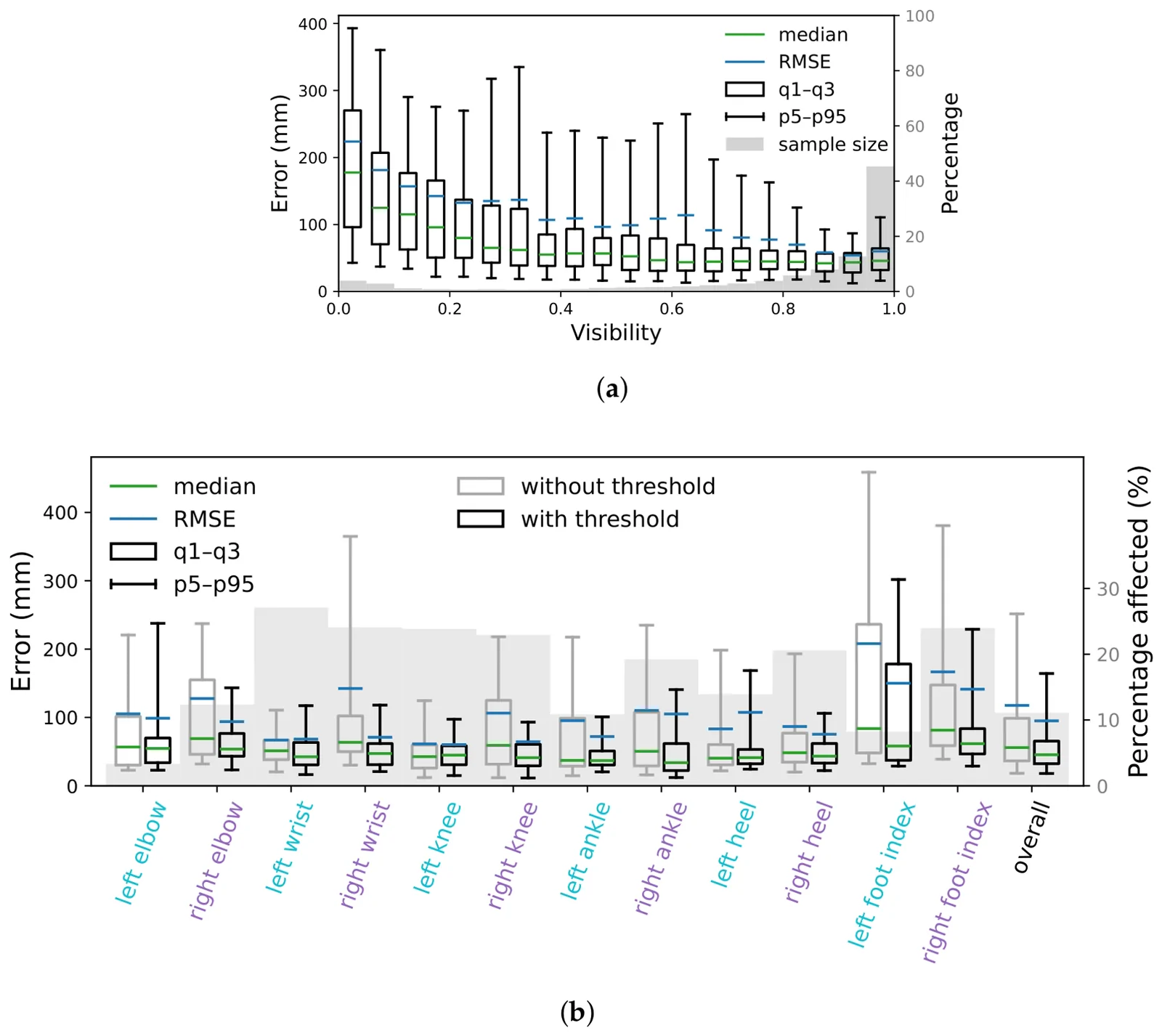
(**a**) Joint position error across visibility bins. (**b**) Comparison of joint position error with and without the visibility threshold for affected observations ($v_{3}<t\leq v_{2}$), where $v_{1}\geq v_{2}\geq v_{3}$ are the ordered camera-view visibility scores and t=0.7. The histogram shows the percentage of affected observations for each joint. Only affected joints are shown.

MediaPipe Pose is sensitive to occlusion and provides a visibility score for each landmark in each frame. For this analysis, joint visibility was defined as the minimum score across all three views at a given time point. Joint position errors between systems in the world frame were pooled across trials and grouped into visibility bins. Errors increased markedly below a visibility of 0.5, and error variance increased below 0.8 (Figure 2a). The distributions for individual joints are shown in Figure S3. Elbow and wrist landmarks frequently had low visibility during the in-line lunge because of occlusion (Figures S1 and S5a). Shoulder and hip landmarks consistently had high visibility because MediaPipe Pose uses them to determine the region of interest before estimating the other landmarks.

#### 3.2.2. Effect of Visibility Threshold on Performance

A visibility threshold reduces the impact of occlusion by excluding camera views with landmark visibility below the threshold. We evaluated thresholds from 0.05 to 0.95 in increments of 0.05 to assess accuracy and data availability, pooling observations across trials and joints except the chest and pelvis (Figure S4). For each joint observation, the three visibility scores were ordered as $v_{1}\geq v_{2}\geq v_{3}$. At threshold *t*, observations were classified as unaffected ($v_{3}\geq t$; all three views used), affected ($v_{3}<t\leq v_{2}$; the lowest-visibility view excluded), or missing ($v_{2}<t$; fewer than two views available). For affected observations, two- and three-view reconstructions were compared on identical observations at each threshold.

Excluding the lowest-visibility view reduced error in affected observations at all evaluated thresholds. Across valid observations, higher thresholds reduced large errors but increased missing data. At a threshold of 0.7, 90.6% of observations remained valid. Raising the threshold to 0.95 reduced the overall 95th-percentile error from 113.7 to 107.6 mm, while retention fell to 69.8%. This balance supported 0.7 as a common operating threshold for this dataset. Joint-specific distributions (Figure S3) do not establish that the score has the same relationship to localization error for every landmark.

At the selected threshold (t=0.7), data availability for each joint and exercise is shown in Figure S5a. For affected observations, excluding the lowest-visibility view reduced joint position error for most joints, especially the third quartile and 95th percentile, although small errors increased for some joints (Figure 2b). Including that view could introduce large 2D estimation errors into the reconstruction. Affected observations had a longer upper error tail than all valid observations, which also included unaffected observations (Figure S5b). Although affected observations represented only 11.1% on average across joints, excluding the low-visibility view markedly reduced the overall 95th-percentile error.

#### 3.2.3. Effect of Camera Configuration on Performance

We compared three two-camera configurations with the three-camera configuration. The proportion of valid observations, requiring joint visibility of at least 0.7 in two or more views, was lower with two cameras, particularly the left–right pair, because of occlusion (Table 1a). Joint position errors were highest for the center–side pairs. The left–right pair had a slightly higher median error than the three-camera configuration but lower RMSE and 95th-percentile error. Thus, the three-camera configuration retained more observations with accuracy comparable to the left–right pair, although the configurations were evaluated on different observations.

Because visibility filtering retained different observations for each camera configuration, the comparison above could reflect differences in observation selection as well as reconstruction accuracy. We therefore evaluated all configurations on identical valid observations, requiring joint visibility of at least 0.7 in all three views (Table 1b). Median, RMSE, and 95th-percentile errors were then lowest with three cameras, slightly higher with the left–right pair, and highest with the center–side pairs. The left–right pair’s lower RMSE and 95th-percentile error in Table 1a therefore partly reflected differences in the retained observations. The three-camera configuration also retained observations with one view below threshold, reconstructing them from the remaining two views. These sometimes involved narrower-angle center–side pairs, which had higher errors. For this camera arrangement, three cameras had the lowest errors when all views had sufficient visibility and retained more observations during occlusion. This comparison does not isolate camera number from angular arrangement.

Joint offset correction (Section 3.3 and Section 3.5) improved overall performance but preserved these qualitative camera-configuration effects (Figures S12 and S13; Table S6). With two cameras, the knee had the highest proportion of missing data, followed by the ankle, heel, and foot index (Figure S12a). Although three cameras produced more large errors than the left–right pair on each configuration’s valid observations (Figure S13a,c,d), their higher multivariate correlations indicated greater overall agreement and structural similarity (Figure S13b).

### 3.3. Systematic Joint Position Offsets Between Systems

Corresponding joint positions from MediaPipe Pose and the marker-based skeletal model can differ systematically because of landmark definitions, as well as measurement and estimation errors. We characterized these differences as joint position offsets in the corresponding body-segment reference frames; the analysis cannot attribute the full offset to the markerless estimator.

#### 3.3.1. Estimation of Joint Position Offset

Each joint was associated with a nearly rigid body segment. A segment reference frame is a local coordinate system that moves with the segment and is aligned with its anatomical axes. We established this frame from markers measured by the marker-based system, using a subject-specific mean marker configuration aligned with an anatomical reference template. Fitting this configuration to the measured marker positions at each time point determined the segment pose. Joint positions from both systems were then transformed into this frame so that they could be compared relative to the segment (Methods Section 2.3.5). For each subject, the systematic joint position offset was measured as the difference between the systems’ mean positions in this frame across all trials (Methods Section 2.4.1).

**Figure 3:**
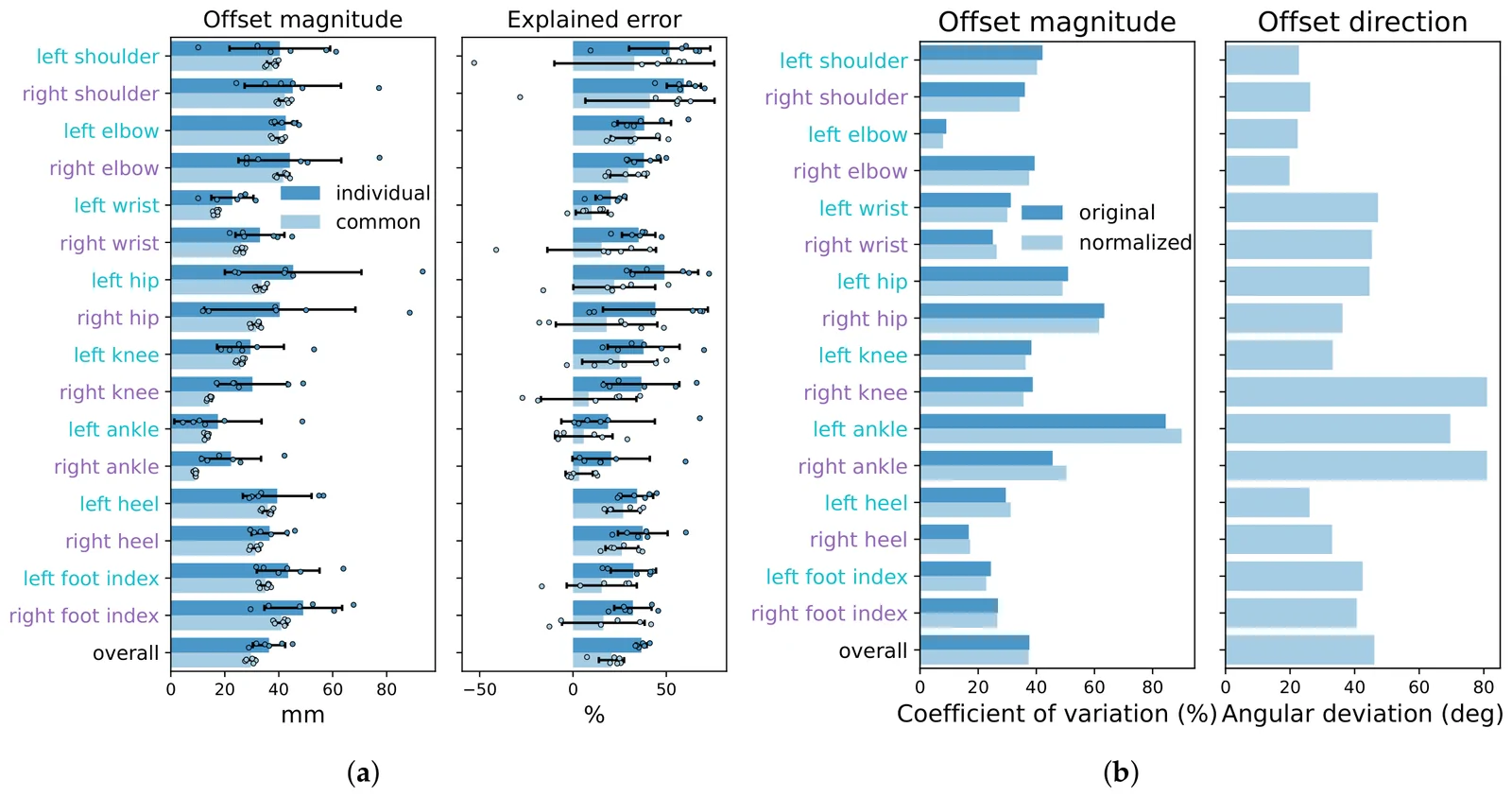
(**a**) Left: Offset magnitude for each joint and subject (individual) and the across-subject average scaled by individual height (common). Right: Percentage of squared joint position disagreement explained by the offset. Dots represent individual subjects. Error bars represent the standard deviation across subjects. (**b**) Joint offset variability across subjects. Left: Coefficient of variation for offset magnitude. Right: Angular deviation of the offset direction in the body-segment reference frame.

To compare offsets across subjects, we normalized each offset to a reference height of 1.70 m and averaged the normalized offsets to obtain a common offset. Figure 3a shows individual offset magnitudes at their original scale and the common offset rescaled to each subject’s height, together with the percentage of squared error explained. On average, individual and common offsets accounted for 36.62% and 20.60% of the total squared error, respectively.

Joint offset components were larger in the medial direction for the shoulders and the lateral direction for the hips (Figure S6 and Table S4). Larger components were also observed in the proximal and anterior directions for the elbows, the distal direction for the heels, and the posterior direction for the foot indices. The heel and foot-index offset directions are consistent with the placement of the physical markers used to represent these landmarks in the marker-based system.

#### 3.3.2. Variability in Joint Offset Across Subjects

For each joint, we calculated the CV of offset magnitude at the original and height-normalized scales and the angular deviation of offset direction in the segment reference frame (Figure 3b). Across all joints, the mean CV of normalized magnitude was 37.33%, and the mean angular deviation was 46.1°. These values describe variability across the six subjects. Inconsistent marker placement may have contributed to angular deviation by affecting the orientation of the segment frames’ anatomical axes.

We also examined the symmetry of joint offsets between the left and right sides across all subjects. Differences in offset magnitude were generally below 20 mm, and differences in direction were generally below 40°. Large angular differences occurred only for offsets of small magnitude (Figure S7).

### 3.4. Variability in Joint Estimation

#### 3.4.1. Directional Patterns of Residual Variability

**Figure 4:**
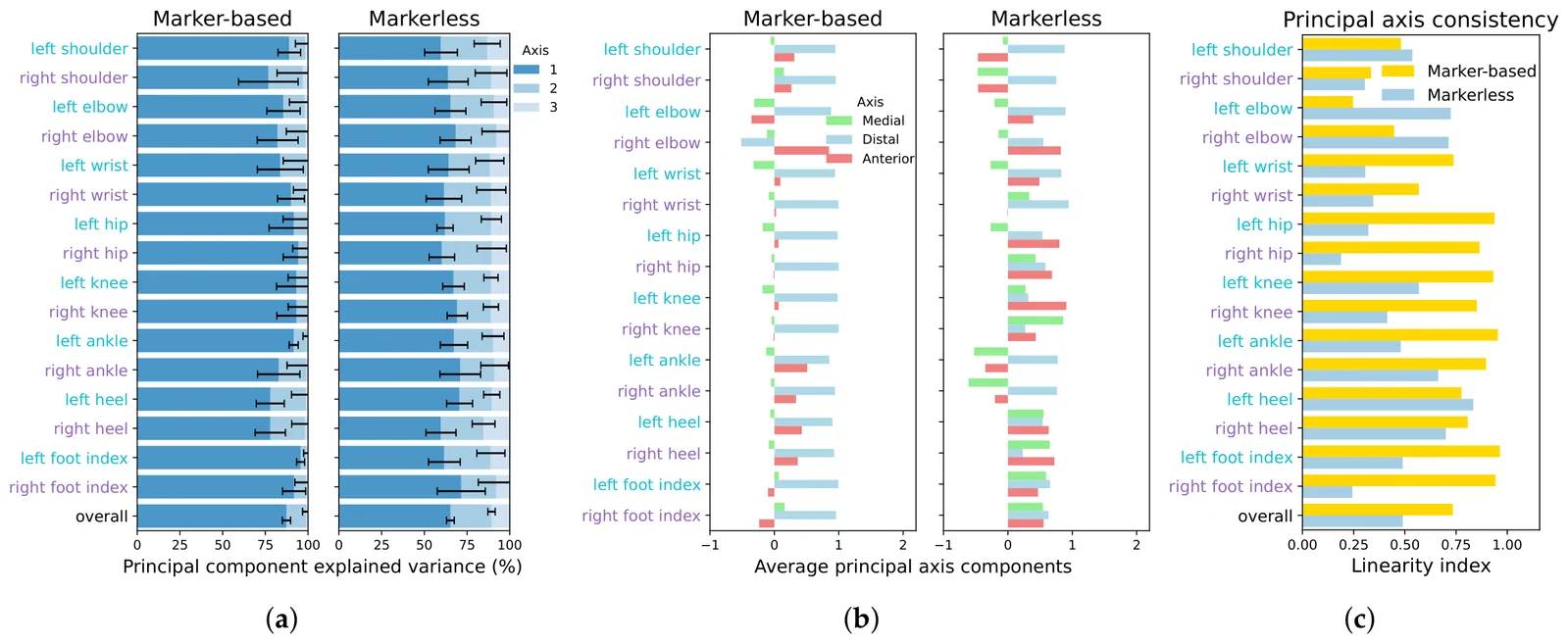
(**a**) Percentage of variance in joint estimates explained by the principal components. Error bars represent the standard deviation across subjects. (**b**) Anatomical-axis components of the mean direction of the first principal axis of joint estimation across subjects. (**c**) Linearity index of the first-principal-axis directions across subjects, indicating directional consistency. A value of 1 indicates perfect parallel alignment, whereas values near 0 indicate little shared directional preference.

After characterizing the systematic joint position offset, we evaluated residual variability in both systems after subtracting the mean position in the segment reference frame. Principal component analysis was performed to obtain the three principal axes for each joint. In the marker-based system, variance was primarily explained by the first component (Figure 4a), which accounted for an average of 87.38%; the first two components together accounted for 99.05%. In the markerless system, the second component explained a somewhat larger proportion of the variance than in the marker-based system. The first component accounted for an average of 65.26%, and the first two components together accounted for 89.35%. These results showed preferential directions of residual variability within individual subjects.

#### 3.4.2. Consistency of Residual Directions Across Subjects

Across subjects, the mean first-principal-axis direction had its largest component along the proximal–distal axis for most joints in the marker-based system; directions were less consistent in the markerless system (Figure 4b). The linearity index measures how closely the first principal axes align across subjects, treating opposite directions as equivalent. The index supported this pattern within the sample (Figure 4c). Analysis of total variance showed a similar pattern: marker-based residual variability was greater along the proximal–distal axis for most joints, whereas markerless residual variability was more uniform across directions (Figure S8).

### 3.5. Performance Metrics

**Figure 5:**
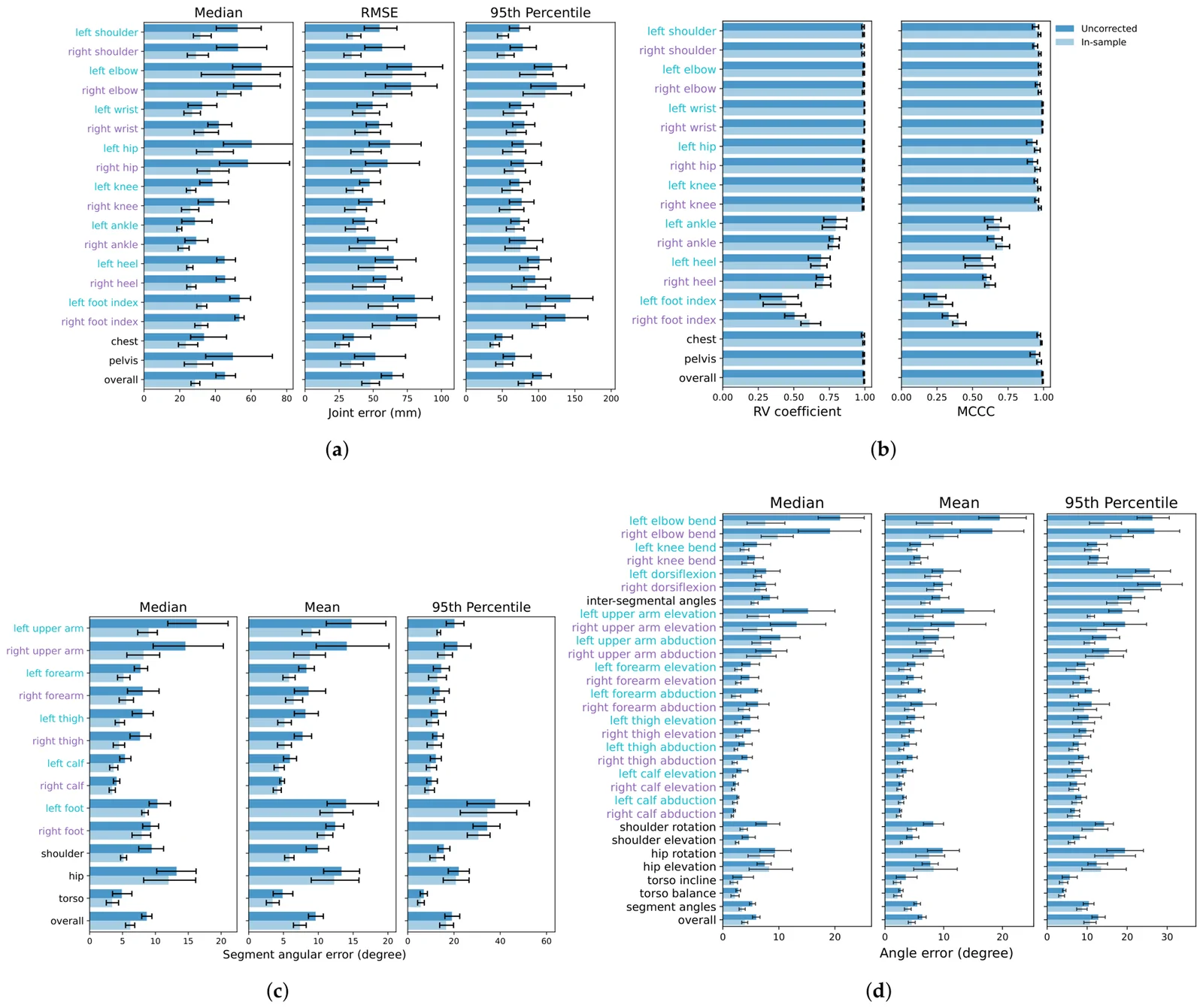
Markerless-system performance without correction (Uncorrected; dark blue) and with in-sample offset correction (In-sample; light blue). (**a**) Joint position error statistics. (**b**) Multivariate correlations: RV-coefficient (left) and matrix-based concordance correlation coefficient (MCCC; right). (**c**) Segment orientation error statistics. (**d**) Inter-segmental and segment angle error statistics. Bars show means across six participants; error bars show 95% participant-bootstrap intervals (Supplementary Section S1).

We evaluated markerless-system performance using marker-based measurements as the reference. Results are presented with and without joint offset correction (Figure 5).

#### 3.5.1. Joint Position Error

Joint position errors in the common world frame were substantially reduced after offset correction (Figure 5a; Table 2). During the in-line lunge, 95th-percentile errors were high for the lunge leg’s knee, ankle, heel, and foot index (Figure S9), indicating occasional tracking failures, particularly during temporary occlusion (Figure S1c,d).

#### 3.5.2. Joint Position Correlation

The RV-coefficient measures structural similarity of joint positions, whereas the matrix-based concordance correlation coefficient (MCCC) measures agreement, including bias and scale. Values close to 1 indicated similar overall 3D position patterns and high agreement between systems. Both metrics improved after joint offset correction (Figure 5b; Table 2).

Low correlations for joints near the feet were attributable to their limited range of motion. Univariate correlation coefficients (Pearson and Lin’s concordance) along each axis are shown in Figure S10. Correlations for the foot joints were low during the deep squat because the feet moved little during this exercise. During the in-line lunge, however, correlations were higher for the foot joints except for the foot index contralateral to the lunge leg, which also moved little. Correlations were particularly high along the Y-axis because movement occurred primarily in that direction (Figure S1c,d).

#### 3.5.3. Segment Orientation and Angle Errors

The segment orientation error is the angle between the segment direction vectors estimated by the two systems. Angle errors measure differences between systems in inter-segmental angles (between adjacent segments) and segment angles (between a segment and an anatomical axis). Both types of error were reduced after joint offset correction (Figure 5c,d; Table 2). Exercise-specific patterns (Figure S11) were consistent with the position errors of the corresponding joints (Figure S9).

#### 3.5.4. Cross-Validation of Offset Correction

The corrected results above represent in-sample performance, with offsets estimated and evaluated using the same trials. Within-subject cross-validation (leave-one-repetition-out) used offsets estimated from a subject’s remaining repetitions to correct one held-out repetition from each trial. Across-subject cross-validation (leave-one-subject-out) used a common offset estimated from the other subjects to correct the held-out subject’s measurements (Methods Section 2.4.3; Table 2).

Within-subject correction yielded position errors, multivariate correlations, and angular errors nearly identical to the in-sample results (Table 2). The mean trial-level median position error was 29.0 mm, compared with 28.6 mm in-sample and 45.4 mm without correction. Across-subject correction improved all performance measures, but gains were smaller, with a mean trial-level median position error of 38.1 mm. Angle errors, particularly inter-segmental angle errors, retained a larger proportion of the in-sample improvement than position errors, multivariate correlations, or segment orientation errors. These results suggest that offsets were stable across repetitions within subjects and partially transferable across subjects when marker-based segment reference frames were available. Because both corrections required marker-based segment reference frames, they characterize offset consistency and transferability rather than a correction applicable to markerless measurements alone.

To assess how well these overall averages represented individual participants and tasks, we also examined participant-level summaries and exercise-specific patterns. Errors varied across participants, and uncorrected mean segment orientation errors were larger during lunges than squats. Individual values, uncertainty estimates, and the supporting procedures are reported in Supplementary Section S1 (Table S8; Figure S14).

## 4. Discussion

This study evaluated a low-cost markerless motion capture system based on three RGB cameras and MediaPipe Pose against a Vicon marker-based reference. During the Functional Movement Screen exercises, joint positions showed high multivariate concordance, and mean segment orientation and overall angle errors were 9.6° and 6.4°, respectively. Calibration residuals were small relative to joint position disagreement, while landmark visibility and systematic offsets affected agreement between systems. For the tested camera arrangement, three cameras improved data availability during occlusion and reconstruction accuracy on identical valid observations. Reference-derived offset correction reduced systematic disagreement. These findings characterize factors affecting agreement with the reference in a lightweight multi-camera markerless motion capture system.

Table S7 summarizes configurations, outcomes, and selected errors from comparable multi-camera studies. Our mean trial-level median joint position error was 45.4 mm without offset correction, and mean overall angle error was 6.4°. Several studies report centimeter-scale position errors, but differences in metrics and protocols prevent direct comparison. For example, Dill et al. [8] reported a median recording-level positional RMSE of 30.9 mm after fitting coordinates to the reference, whereas our uncorrected position errors were evaluated after a global mapping derived from calibration-board measurements. Ceriola et al. [10] calculated sagittal hip and knee waveform RMSE after removing cycle-level angle offsets. OpenCap [11] reported a mean absolute error (MAE) of 4.5° for model-based joint rotations using HRNet, after anatomical-marker augmentation and constrained inverse kinematics. These results provide context for the magnitude of the observed errors rather than a performance ranking across systems. They motivate further evaluation of lightweight multi-camera systems for clinical assessment, sports science, and rehabilitation, although our small sample limits conclusions about suitability across populations and tasks.

Practical use requires matching measurement precision to the intended outcome. Centimeter-scale joint position disagreement may be tolerable for gross movement tracking, but it does not support precise anatomical joint-center localization. A mean overall angle error of 6.4° also warrants caution for outcomes that depend on changes of only a few degrees. Functional screening requires validation of the relevant scores or decisions; the present study did not validate movement classification, Functional Movement Screen scoring, or a minimum detectable change.

The small calibration residuals relative to joint position errors suggest that calibration refinement alone would yield limited improvements in reconstruction accuracy. For the assumed one-frame inter-camera timing difference, the displacement analysis indicated millimeter-scale movement at the median joint speed but centimeter-scale movement during faster phases (Table S3). Temporal misalignment could therefore contribute to position error, although its actual contribution remains uncertain because timing differences were not independently measured. Improving landmark detection remains important, alongside verifying synchronization during movement.

The camera configuration comparisons highlight the combined effects of landmark visibility and camera geometry. In our setup, an additional view increased joint availability and improved robustness during occlusion. Bajrami et al. [9] reported task-dependent differences between two- and three-view reconstructions, while Samani et al. [12] found that angular agreement varied across camera pairs. These findings support evaluating camera geometry alongside detection robustness; additional views or wider separation do not necessarily reduce error.

Multi-camera precision also depends on camera number and angular arrangement, as modeled by Yin et al. [13]. On identical valid observations (Table 1b), the left–right pair (75°) outperformed the center–side pairs (37.5°), consistent with stronger geometric constraints for comparable 2D landmark uncertainty [18]. The third camera provided a small additional accuracy improvement as well as greater availability during occlusion in this setup. These findings apply to the tested positions and tasks and do not establish a camera-number advantage independent of angular arrangement. Although matching controlled differences in observation availability, geometric and landmark-localization contributions were not independently quantified because 2D localization uncertainty was not characterized.

Excluding low-visibility detections reduced large reconstruction errors. A threshold of 0.7 balanced accuracy and availability, as higher thresholds yielded little further reduction in large errors but substantially increased missing data (Figure S4). Visibility is a model score, not a calibrated probability of correct localization, and its relationship to error may differ among joints and camera views. The pooled threshold sweep can also be influenced more by frequently observed or difficult landmarks. Hard rejection was used to characterize the implemented DLT pipeline; retained views were equally weighted. Continuous confidence weighting could retain useful information from views just below the threshold and alter the accuracy–availability trade-off, but this was not tested. This possibility is consistent with Fortini et al.’s reconstruction framework [14], which combines confidence-threshold rejection with continuous weighting based on confidence, distance, and body orientation.

Systematic joint position offsets were observed for several landmarks. When offsets were defined in local segment reference frames using marker-based measurements, subject-specific correction reduced joint position disagreement. On average, subject-specific offsets accounted for 36.62% of the total squared difference, whereas a common offset applied across subjects accounted for 20.60%, indicating variability across subjects. Because both the local segment reference frames and joint offsets were derived from marker-based measurements, this correction cannot be applied directly to markerless data alone. Within-subject cross-validation yielded results close to the in-sample correction, whereas leave-one-subject-out validation yielded smaller reductions (Table 2). The latter assesses transfer of offset patterns when the held-out participant’s marker-based segment frames are available. Estimating those frames from markerless geometry and repeating leave-one-subject-out validation is a necessary next step toward standalone correction.

The observed offsets may arise from differences between the anatomical landmarks estimated by MediaPipe Pose and the joint positions defined by the marker-based skeletal model, as well as from pose-estimator training labels. Marker placement, soft-tissue motion, clothing, image quality, and reference-frame estimation may also contribute. The present measurements do not separate these contributions or establish the anatomical correctness of either system. Future pose estimation algorithms may benefit from anatomical and temporal constraints. For example, Wei et al. [26] combined limb-length invariance, bilateral symmetry, and motion continuity to improve 3D pose estimation, suggesting a direction for improving joint estimates without reference-derived correction during use.

After subtracting mean joint positions in each segment frame, residual variability showed preferential directions within individual subjects. These directions were less consistent across subjects in the markerless system than in the marker-based system. Marker-based variability was primarily proximal–distal for most joints, whereas markerless variability was distributed more uniformly across anatomical directions. These residuals may contain soft-tissue motion, segment deformation, marker-placement effects, reference-frame estimation error, and pose-dependent systematic differences. They therefore describe residual variability rather than a demonstrated random-error process.

Downstream kinematic measures remained comparatively stable despite centimeter-scale joint position errors. Segment orientation errors followed the position-error patterns of their associated joints, as expected because each segment direction is determined by its joint locations. Although inter-segmental angles depend on two adjacent segment orientations, their errors were comparable in magnitude to individual segment orientation errors rather than increasing through their combination. Offset correction reduced position, segment orientation, and angle errors by comparable proportions, indicating that reducing systematic joint position disagreement can also reduce angular disagreement. When offsets were estimated from other subjects, angle errors retained a larger proportion of the in-sample improvement, particularly for inter-segmental angles, than position or segment orientation errors (Table 2). The benefit from transferring offset correction therefore depended on the kinematic outcome.

Several limitations should be considered when interpreting these results. Six healthy adults performed two Functional Movement Screen exercises in a controlled indoor environment. The analyses describe error magnitudes and variability within the recorded dataset and do not provide confirmatory evidence of population-level statistical significance. Six independent participants provide limited precision for population comparisons, and repeated frames or trials do not increase that independent sample. Participant-bootstrap intervals are exploratory and do not overcome this limitation (Supplementary Section S1). The small sample also limits the generalizability of the observed offset and residual-variability patterns across subjects. Leave-one-subject-out cross-validation assesses transferability within this cohort, but broader conclusions require a larger, more diverse sample. Further evaluation of clinical populations, faster or predominantly upper-body movements, other asymmetric tasks, and less controlled conditions, including outdoor settings and varied clothing or lighting, is needed to establish suitability for clinical, sports, and rehabilitation applications. Restricted motion, altered posture, and loose clothing may affect landmark visibility and localization and alter systematic offsets, so performance should be evaluated against application-specific accuracy requirements. Only MediaPipe Pose and one camera arrangement were evaluated; other estimators, camera placements, and hardware configurations may improve performance.

Synchronization and processing also limit interpretation. The RGB cameras were grouped by acquisition loop without a shared hardware trigger. Only a common timestamp for each three-frame set was retained, so individual-camera timing offsets and jitter could not be quantified. Displacement estimates therefore depend on the assumed inter-camera timing difference, particularly during faster movements. Residual temporal misalignment between systems was also not independently measured. Retrospective lag selection using evaluated joints may make agreement optimistic; the sensitivity check does not eliminate this effect (Supplementary Section S2). Although cameras operated at 30 FPS, MediaPipe Pose processing of the three camera streams ran at approximately 20 FPS on the PC used, limiting real-time feedback frequency. Offline analysis retained the acquisition timestamps, so processing throughput did not determine the sampling rate of the evaluated trajectories. End-to-end latency was not measured, so the reported spatial errors alone do not establish suitability for real-time feedback.

In conclusion, this study characterizes how landmark visibility, camera configuration, and systematic joint offsets affect reconstructed positions and downstream kinematics in six healthy adults performing deep squats and in-line lunges with the tested camera arrangement. Within this cohort, cross-validation describes offset consistency within subjects and partial transferability across subjects when marker-based segment reference frames are available.

## Figures and Tables

**Table 1:** Overall joint position error statistics (mm) and percentage of valid observations (%) across all joints under different camera configurations. (**a**) Each configuration’s valid observations. (**b**) Identical valid observations: time points at which all three cameras detected the participant and joint visibility was ≥0.7 in every view. The percentage of valid observations in (**b**) is therefore the same for all configurations. Values are reported as the mean (SD) across trials.

	Median	RMSE	95th Percentile	Visible Percentage
(**a**) Valid observations				
All	45.4 (7.6)	64.2 (15.1)	104.5 (25.0)	91.05 (5.93)
Left–right	45.8 (9.6)	61.9 (13.1)	100.9 (22.7)	81.41 (12.59)
Center–left	49.5 (7.8)	73.7 (16.1)	122.9 (29.1)	83.88 (10.56)
Center–right	50.1 (8.0)	75.1 (19.1)	123.7 (29.9)	86.62 (8.84)
(**b**) Identical valid observations				
All	45.6 (8.5)	59.3 (12.9)	98.8 (25.2)	80.43 (13.01)
Left–right	46.0 (9.9)	61.9 (13.6)	100.9 (23.4)	
Center–left	49.9 (8.0)	73.3 (15.7)	122.8 (29.4)	
Center–right	50.2 (8.6)	72.5 (18.0)	120.5 (31.8)	

**Table 2:** Overall performance without correction (Uncorrected), with subject-specific in-sample correction (In-sample), subject-specific leave-one-repetition-out correction (Within-subject), and common leave-one-subject-out correction (Across-subject). Values are the mean (sample SD) across six participants after averaging trial statistics within participants (Supplementary Section S1). Within-trial statistics pool data across joints or average errors across segments and angle metrics, as described in Methods. In SD rows, the statistic measures within-trial dispersion; parentheses give between-participant SD. Corrected results exclude the 4.1% of joint observations with unavailable marker-based segment frames.

	Uncorrected	In-Sample	Within-Subject	Across-Subject
Joint position error (mm)				
Median	45.4 (7.6)	28.6 (3.5)	29.0 (3.6)	38.1 (7.6)
RMSE	64.2 (11.0)	48.0 (9.0)	48.5 (9.2)	56.4 (10.7)
95th percentile	104.5 (17.6)	81.0 (12.7)	81.7 (12.7)	92.4 (16.0)
Multivariate correlation				
RV-coefficient	0.9934 (0.0049)	0.9954 (0.0043)	0.9953 (0.0044)	0.9944 (0.0046)
MCCC	0.9925 (0.0052)	0.9949 (0.0044)	0.9949 (0.0045)	0.9933 (0.0049)
Segment orientation error (°)				
Mean	9.6 (1.5)	7.4 (1.2)	7.4 (1.3)	8.5 (1.6)
SD	5.3 (1.4)	5.0 (1.4)	5.1 (1.4)	5.0 (1.4)
Median	8.7 (1.1)	6.2 (0.9)	6.3 (0.9)	7.5 (1.4)
95th percentile	19.1 (4.3)	16.6 (4.0)	16.7 (4.0)	17.8 (4.1)
Inter-segmental angle error (°)				
Mean	9.4 (1.9)	6.9 (1.1)	7.0 (1.1)	7.4 (1.7)
SD	6.2 (1.4)	5.5 (1.4)	5.5 (1.4)	5.5 (1.3)
Median	8.4 (1.9)	5.7 (0.9)	5.7 (0.9)	6.4 (1.5)
95th percentile	21.2 (4.6)	17.8 (4.0)	17.8 (4.0)	18.2 (4.5)
Segment angle error (°)				
Mean	5.5 (0.8)	3.9 (0.8)	3.9 (0.8)	4.6 (0.9)
SD	3.0 (0.6)	2.7 (0.6)	2.7 (0.6)	2.7 (0.6)
Median	5.3 (0.7)	3.4 (0.8)	3.5 (0.7)	4.1 (0.8)
95th percentile	10.4 (1.9)	8.7 (1.8)	8.8 (1.8)	9.4 (1.8)
Overall angle error (°)				
Mean	6.4 (0.9)	4.6 (0.8)	4.6 (0.8)	5.2 (0.9)
SD	3.7 (0.7)	3.3 (0.7)	3.3 (0.7)	3.4 (0.6)
Median	6.0 (0.9)	3.9 (0.7)	4.0 (0.7)	4.6 (0.8)
95th percentile	12.8 (2.3)	10.7 (2.1)	10.8 (2.2)	11.3 (2.1)

## Data Availability

The data supporting the findings of this study are available from the corresponding author upon reasonable request. The sharing of image and video recordings is subject to each participant’s specific consent and applicable privacy and institutional requirements; therefore, recordings from participants who did not consent to such sharing cannot be provided.

## Supplementary Materials

The following supporting information can be downloaded at https://www.mdpi.com/article/10.3390/s26216773/s1. Section S1: Participant-level variability and uncertainty; Section S2: Sensitivity to inter-system alignment. Figure S1: Exercises from the Functional Movement Screen [15]. (**a**,**b**) Deep squat. (**c**,**d**) Left-leg in-line lunge. Panels (**a**) and (**c**) show center-camera views; panels (**b**) and (**d**) show side-camera views. The right-leg lunge was performed in the opposite direction. Figure S2: Front view of the eight checkerboard poses in the world reference frame, corresponding to Figure 1c. ‘Ground truth’ in the legend denotes ideal corner coordinates defined by the measured board dimensions. Table S1: Error statistics for checkerboard corner points after transformations between reference frames, with data pooled across all sessions (mm). Table S2: Scaling bias of similarity transformations between reference frames (%). Values are reported for each recording session and as the mean absolute scaling bias across sessions. Table S3: Joint speeds and estimated displacements for an assumed inter-camera timing difference of one frame interval (33 ms at 30 FPS). Timing differences were not independently measured. Displacements were calculated by multiplying marker-based joint speeds by this interval. “Pooled” reports the 95th percentile across trials; “Trials” reports the mean (SD) of trial-specific 95th percentiles. “All joints” additionally pools joints. The median speed across all joints was 0.07 m/s, corresponding to 2.3 mm. These values estimate movement between captures rather than reconstruction error, which also depends on camera arrangement and motion direction. Figure S3: Joint position error distributions across visibility bins for individual joints. Figure S4: Sensitivity of joint position error and data availability to the visibility threshold. For each joint observation, camera-view visibility scores are ordered as $v_{1}\geq v_{2}\geq v_{3}$. At threshold *t*, observations are unaffected ($v_{3}\geq t$; all three views used), affected ($v_{3}<t\leq v_{2}$; lowest-visibility view excluded), or missing ($v_{2}<t$; fewer than two views available). Data are pooled across trials and joints, excluding the chest and pelvis. Top: Errors for affected observations reconstructed using three views (gray) or the two highest-visibility views (black), evaluated on identical observations at each threshold. Middle: Errors for all valid observations after filtering; the retained observations change with the threshold. Bottom: Percentages of unaffected (dark blue) and affected (light blue) observations relative to all observations with marker-based data. The gap to the dashed 100% line represents missing observations. The selected threshold of 0.7 is highlighted. Figure S5: (**a**) Data availability in each trial for every joint and exercise: marker-based observations present (yellow) and markerless observations reconstructed with visibility ≥0.7 (blue). (**b**) Joint position error with and without visibility filtering on identical valid observations ($v_{2}\geq t$), pooled across trials. Camera-view scores are ordered as $v_{1}\geq v_{2}\geq v_{3}$, and t=0.7. Both affected ($v_{3}<t\leq v_{2}$) and unaffected ($v_{3}\geq t$) observations are included. Figure S6: Components of the mean joint offset at the original scale along the anatomical axes. Error bars represent the standard deviation across subjects. Table S4: Joint offset components along the anatomical axes (mm), normalized to a reference height of 1.70 m. Values are reported as the mean (SD) across subjects. Positive components indicate medial, distal, and anterior directions; negative components indicate lateral, proximal, and posterior directions, respectively. Figure S7: Differences in joint offset magnitude and direction between the left and right sides for all subjects. Marker size represents the mean offset magnitude across both sides. Figure S8: Residual variability of joint positions in the segment reference frame, expressed as the standard deviation along each anatomical axis. Error bars represent the standard deviation across subjects. Figure S9: Joint position error statistics after offset correction for each exercise. Error bars show 95% bootstrap confidence intervals across six participants. Figure S10: Pearson correlation coefficients and Lin’s concordance correlation coefficients for offset-corrected joint positions along each world axis. (**a**) Deep squat. (**b**) In-line lunge (left). (**c**) In-line lunge (right). Error bars show 95% bootstrap confidence intervals across six participants. Table S5: Overall joint position correlations along each world axis, pooling all joints within each trial. Pearson correlation coefficients (PCCs) and Lin’s concordance correlation coefficients (CCCs) are reported as the mean (SD) across trials. “Original” denotes uncorrected estimates; “Offset” denotes offset-corrected estimates. Figure S11: Segment orientation error statistics after offset correction for each exercise: (**a**) deep squat and (**b**) in-line lunge. Error statistics for inter-segmental and segment angles after offset correction: (**c**) deep squat and (**d**) in-line lunge. All error bars show 95% bootstrap confidence intervals across six participants. Figure S12: Markerless data availability for each exercise under different camera configurations. “All” (blue) denotes three cameras; the other labels (gray) denote camera pairs. (**a**) Percentage of valid observations for each joint (visibility ≥0.7). (**b**) Percentage of each trial in which all joints required for the metrics were simultaneously available. Error bars show 95% bootstrap confidence intervals across six participants. Figure S13: Offset-corrected markerless-system performance on each camera configuration’s valid observations. “All” (blue) denotes three cameras; the other labels (gray) denote camera pairs. (**a**) Joint position error statistics. (**b**) Multivariate correlations for joint positions: RV-coefficient (left) and matrix-based concordance correlation coefficient (MCCC; right). (**c**) Segment orientation error statistics. (**d**) Error statistics for inter-segmental and segment angles. Bars show means and 95% bootstrap confidence intervals across six participants (Section S1). Table S6: Overall offset-corrected joint position error statistics (mm) on each camera configuration’s valid observations, pooling all joints within each trial. Values are reported as the mean (SD) across trials. Table S7: Comparison of selected multi-camera markerless motion capture studies. (**a**) System configurations and validation protocols. Comparison of selected multi-camera markerless motion capture studies, continued. (**b**) Selected positional and angular errors. Figure S14: Uncorrected joint position, segment orientation, and overall angle errors across participants and exercise conditions. Colored points and lines identify participants; black squares and error bars show cohort means and 95% participant-bootstrap intervals. Joint position errors are trial-level medians; segment orientation and overall angle errors are trial-level means. Table S8: Joint position, segment orientation, and overall angle errors for six participants, using equal trial weights within participants. Joint position errors average trial-level medians; segment orientation and overall angle errors average trial-level means. The final columns give the mean (sample SD) and 95% participant-bootstrap interval. Correction schemes follow manuscript Table 2. Figure S15: Mean paired changes in uncorrected errors from the quadratic zero-shift baseline, using identical observations across all six conditions. Error bars show 95% participant-bootstrap intervals; the dashed line marks no change. One frame is the recording-specific median markerless interval (32.63–36.18 ms). References cited in the Supplementary Materials: [6,8,9,10,11,12,15].

## Abbreviations

   The following abbreviations are used in this manuscript:

CCC	Concordance correlation coefficient
CV	Coefficient of variation
DLT	Direct linear transformation
FPS	Frames per second
GPA	Generalized Procrustes analysis
MCCC	Matrix-based concordance correlation coefficient
PCA	Principal component analysis
PCC	Pearson correlation coefficient
RGB	Red, green, and blue
RMSE	Root mean square error

## Appendix A. Generalized Procrustes Analysis

Let the marker positions in the segment reference template be the sequence $x^{*}={\left(x_{i}^{*}\right)}_{i=1}^{n}$, and let their positions in the world frame at the *j*-th of *m* time points be $y_{j}={\left(y_{ij}\right)}_{i=1}^{n}$. Given a mean configuration $x={\left(x_{i}\right)}_{i=1}^{n}$, define the rigid transformation $T_{j}\in \mathrm{SE}\left(3\right)$ from the world frame to the segment frame at time *j* as follows:

(A1) $$T_{j}\left(x\right)=\underset{T}{\mathrm{argmin}}\sum_{i=1}^{n}{∥Ty_{ij}-x_{i}∥}^{2}$$

There are an infinite number of possible mean configurations, represented by the set

(A2) $$X=\{x\;|\;x_{i}=\frac{1}{m}\sum_{j=1}^{m}T_{j}\left(x\right)y_{ij},\;\mathrm{for}\;i=1,2,\ldots ,n\}$$

We seek a unique solution that best aligns with the reference template while preserving its orientation relative to the anatomical axes:

(A3) $$\hat{x}=\underset{x\in X}{\mathrm{argmin}}\sum_{i=1}^{n}{∥x_{i}-x_{i}^{*}∥}^{2}$$

The optimization problem can be solved using the following alternating optimization procedure (Algorithm A1):

**Algorithm A1** Generalized Procrustes Analysis
**Require:** Marker data $y_{j}$ for j=1,2,…,m; reference template $x^{*}={\left(x_{i}^{*}\right)}_{i=1}^{n}$ **Ensure:** Optimized mean configuration $x=\hat{x}$; rigid transformations $T_{j}=T_{j}\left(\hat{x}\right)$ for all *j* 1: $x\leftarrow x^{*}$ ▹ Initialize mean configuration from reference template 2: **repeat** 3: $x_{\mathrm{prev}}\leftarrow x$ 4: $T_{j}\leftarrow T_{j}\left(x\right)$ for all *j* ▹ Compute rigid transformations using Equation (A1) 5: $x_{i}\leftarrow \frac{1}{m}\sum_{j=1}^{m}T_{j}\left(x\right)y_{ij}$ for all *i* ▹ Update mean configuration 6: $T_{\mathrm{ref}}\leftarrow {\mathrm{argmin}}_{T}\sum_{i=1}^{n}{∥Tx_{i}-x_{i}^{*}∥}^{2}$ 7: $x_{i}\leftarrow T_{\mathrm{ref}}x_{i}$ for all *i* ▹ Align the mean configuration with the reference template 8: $d=\max_{i}\left(∥x_{i}-x_{\mathrm{prev}\;i}∥\right)/\max_{i}\left(∥x_{i}∥\right)$ ▹ Relative change for the current iteration 9: **until** d<tolerance or the maximum number of iterations has been reached 10: $T_{j}\leftarrow T_{\mathrm{ref}}T_{j}$ for all *j* ▹ Compose transformations **return** x, $T_{j}$ for j=1,2,…,m

**Table A1:** Reference templates for body segments. Joints associated with each segment are listed in the header rows. Marker coordinates in the segment reference frame are provided along the anatomical axes in millimeters. The template coordinates are symmetric between the left and right sides. The coordinate axes follow the right-hand rule on the left side; after the medial axis is mirrored, they follow the left-hand rule on the right side.

Marker Description	Medial	Distal	Anterior
Forearm—Joint: wrist			
Ulnar styloid	25	0	0
Radial styloid	−25	0	0
5 cm proximal	25	−50	0
4 cm proximal	−25	−40	0
Forearm marker	0	−100	0
Virtual elbow joint center *	0	−220	0
Upper arm—Joint: shoulder, elbow			
Virtual elbow joint center *	0	0	0
Lateral epicondyle	−30	0	0
Medial epicondyle	30	0	0
Lateral distal	40	−70	10
Lateral proximal	40	−140	20
Posterior distal	0	−60	−20
Posterior proximal	0	−120	−25
Virtual shoulder joint center *	0	−260	0
Thigh—Joint: hip, knee			
Virtual knee joint center *	0	0	0
Lateral knee marker	−45	0	0
Thigh wand (lateral/anterior)	−50	−240	30
Virtual hip joint center *	0	−400	0
Calf—Joint: ankle			
Virtual ankle joint center *	0	0	0
Lateral malleolus	−35	0	0
Tibia wand marker	−50	−180	0
Lateral knee marker	−45	−320	0
Virtual knee joint center *	0	−320	0
Foot—Joint: heel, foot index			
Virtual ankle joint center *	0	0	0
Lateral malleolus	−35	0	0
Heel marker	0	−40	−30
Toe marker	0	120	−20

* Virtual markers are nonphysical markers derived from the skeletal model.

## Appendix B. Statistical Analysis

### Appendix B.1. Linearity Index

The linearity index ($L_{3}$) is a normalized scalar metric that quantifies the directional consistency of 3D axial data by measuring how tightly a set of lines clusters along a single principal axis [27]. The analysis is based on the normalized 3×3 orientation tensor T, defined for a data set of *N* unit vectors $v_{i}$ as follows:

(A4) $$T=\frac{1}{N}\sum_{i=1}^{N}v_{i}v_{i}^{T}$$

The index scales the largest eigenvalue ($\lambda_{1}\geq \lambda_{2}\geq \lambda_{3}$) of T relative to its isotropic baseline of 1/3; an index value of 1 represents perfect parallel alignment, whereas a value of 0 indicates an isotropic configuration with no preferred axis [22]. For small samples, the unbiased estimator of the linearity index is given by [28]

(A5) $$L_{3,\;\mathrm{unbiased}}=\max\left(0,\frac{3N\lambda_{1}-N-2}{2(N-1)}\right)$$

### Appendix B.2. Univariate Correlation Coefficients

Given two univariate random variables *x* and *y*, the Pearson correlation coefficient is given by

(A6) $$\rho \left(x,y\right)=\frac{\mathrm{cov}(x,y)}{\sigma_{x}\sigma_{y}}$$

and Lin’s concordance correlation coefficient is given by

(A7) $$\rho_{C}\left(x,y\right)=\frac{2\rho \left(x,y\right)\sigma_{x}\sigma_{y}}{\sigma_{x}^{2}+\sigma_{y}^{2}+{\left(\mu_{x}-\mu_{y}\right)}^{2}}$$

where $\mu_{x}$ and $\mu_{y}$ are the means of *x* and *y*, $\sigma_{x}$ and $\sigma_{y}$ are the standard deviations, and cov(x,y) is the covariance between *x* and *y*.

### Appendix B.3. Multivariate Correlation Coefficients

Given two multivariate random variables *X* and *Y*, the RV-coefficient is given by

(A8) $$\mathrm{RV}\left(X,Y\right)=\frac{\mathrm{tr}(\Sigma_{XY}\Sigma_{YX})}{\sqrt{\mathrm{tr}\left(\Sigma_{XX}^{2}\right)\mathrm{tr}\left(\Sigma_{YY}^{2}\right)}}$$

and the matrix-based concordance correlation coefficient is given by

(A9) $$\rho_{c,m}\left(X,Y\right)=\frac{2\;\mathrm{tr}(\Sigma_{XY})}{\mathrm{tr}\left(\Sigma_{XX}\right)+\mathrm{tr}\left(\Sigma_{YY}\right)+{∥\mu_{X}-\mu_{Y}∥}^{2}}$$

where $\mu_{X}$ and $\mu_{Y}$ are the mean vectors of *X* and *Y*, respectively; $\Sigma_{AB}$ is the covariance or cross-covariance matrix of *A* and *B* for A,B∈{X,Y}; and tr(·) denotes the matrix trace.
