# Supplementary material for: Evaluation and Error Characterization of a Multi-View 3D Markerless Motion Capture System for Human Kinematics
Source: Sensors (Basel). 2026 Oct 26;26(21):6773. doi: 10.3390/s26216773 (PMC13766164; doi:10.3390/s26216773)
Supplement: Supplementary file 1 [file sensors-26-06773-s001.zip › sensors-4509022-supplementary.pdf]

Supplementary Materials: Evaluation and Error Characterization of a Multi-View 3D Markerless Motion Capture System for Human Kinematics

Ziao Chen [1], Francisco Espinoza [2], Sajeda Al-Hammouri [3,4], Christopher J. Arellano [2,3] and Kavan Hazeli [5,*]

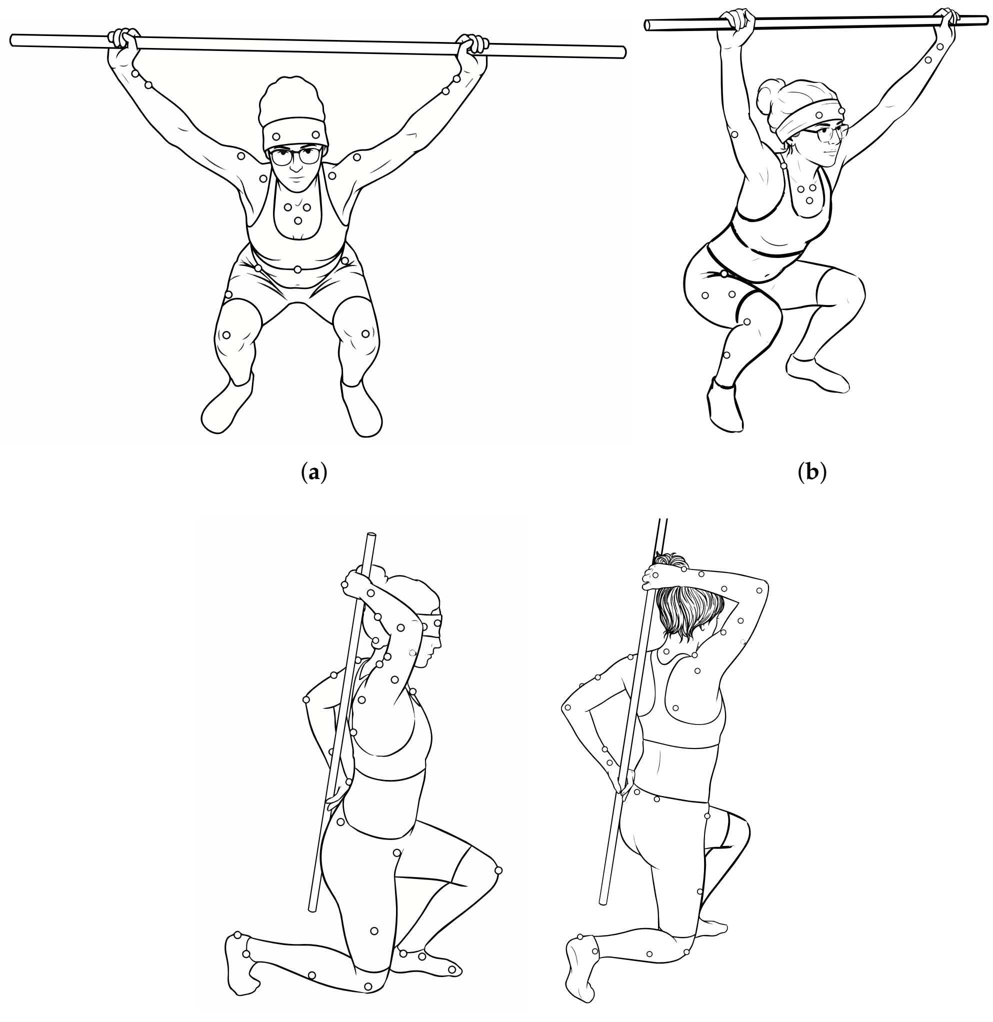

(**a**) (**b**) (**c**) (**d**)

Figure S1. Exercises from the Functional Movement Screen [1]. (**a**,**b**) Deep squat. (**c**,**d**) Left-leg in-line lunge. Panels (**a**) and (**c**) show center-camera views; panels (**b**) and (**d**) show side-camera views. The right-leg lunge was performed in the opposite direction.

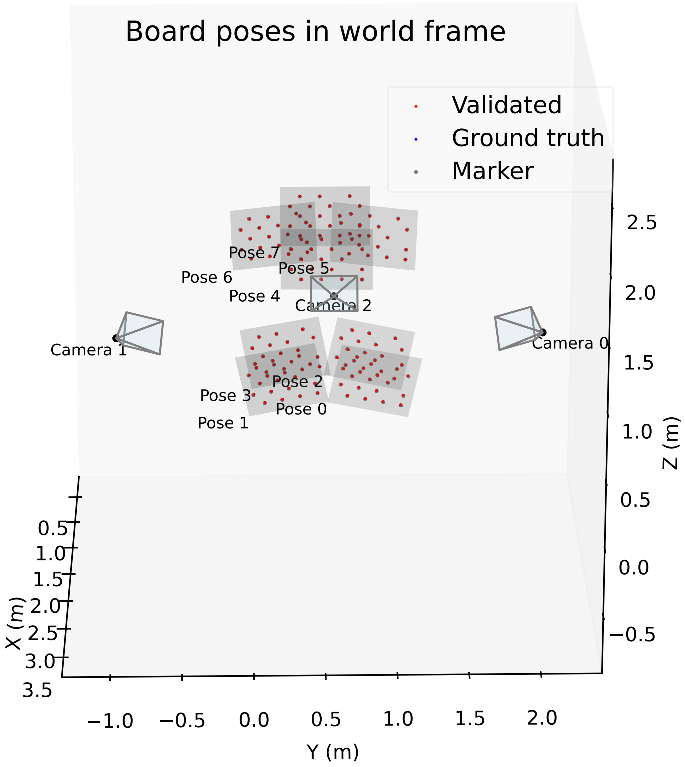

Figure S2. Front view of the eight checkerboard poses in the world reference frame, corresponding to Figure 1c. 'Ground truth' in the legend denotes ideal corner coordinates defined by the measured board dimensions.

Table S1. Error statistics for checkerboard corner points after transformations between reference frames, with data pooled across all sessions (mm).

Transform	Median	RMSE	95th percentile
Board–World	1.21	1.49	2.74
Board–Rig	0.81	1.28	2.63
Rig–World (local)	1.42	1.71	3.11
Rig–World (global)	1.65	2.97	6.23

Table S2. Scaling bias of similarity transformations between reference frames (%). Values are reported for each recording session and as the mean absolute scaling bias across sessions.

Transform	Session 1	Session 2	Session 3	Mean absolute bias
Board–World	−0.25	−0.21	−0.33	0.26
Board–Rig	1.20	−0.11	−0.10	0.47
Rig–World (local)	−1.42	−0.10	−0.22	0.58
Rig–World (global)	−2.32	0.18	0.20	0.90

Table S3. Joint speeds and estimated displacements for an assumed inter-camera timing difference of one frame interval (33 ms at 30 FPS). Timing differences were not independently measured. Displacements were calculated by multiplying marker-based joint speeds by this interval. "Pooled" reports the 95th percentile across trials; "Trials" reports the mean (SD) of trial-specific 95th percentiles. "All joints" additionally pools joints. The median speed across all joints was 0.07 m/s, corresponding to 2.3 mm. These values estimate movement between captures rather than reconstruction error, which also depends on camera arrangement and motion direction.

	Speed (m/s)		Displacement (mm)	
**Joint**	**Pooled**	**Trials**	**Pooled**	**Trials**
Left shoulder	0.65	0.63 (0.11)	21.5	21.1 (3.6)
Right shoulder	0.64	0.64 (0.11)	21.3	21.2 (3.6)
Left elbow	0.68	0.67 (0.13)	22.8	22.4 (4.4)
Right elbow	0.67	0.67 (0.13)	22.2	22.4 (4.2)
Left wrist	0.72	0.70 (0.14)	24.2	23.5 (4.6)
Right wrist	0.71	0.68 (0.15)	23.6	22.7 (5.0)
Left hip	0.59	0.59 (0.14)	19.8	19.6 (4.6)
Right hip	0.59	0.59 (0.14)	19.7	19.6 (4.6)
Left knee	0.66	0.60 (0.22)	22.1	20.1 (7.3)
Right knee	0.72	0.65 (0.26)	24.0	21.8 (8.7)
Left ankle	0.45	0.57 (0.65)	15.0	19.0 (21.5)
Right ankle	0.71	0.69 (0.71)	23.8	23.0 (23.5)
Left heel	0.52	0.63 (0.67)	17.4	20.9 (22.2)
Right heel	0.67	0.72 (0.73)	22.3	24.0 (24.3)
Left foot index	0.40	0.52 (0.73)	13.2	17.2 (24.3)
Right foot index	0.62	0.66 (0.78)	20.5	22.1 (26.0)
Chest	0.64	0.63 (0.11)	21.2	21.0 (3.6)
Pelvis	0.59	0.59 (0.14)	19.7	19.6 (4.6)
All joints	0.65	0.65 (0.14)	21.7	21.7 (4.6)

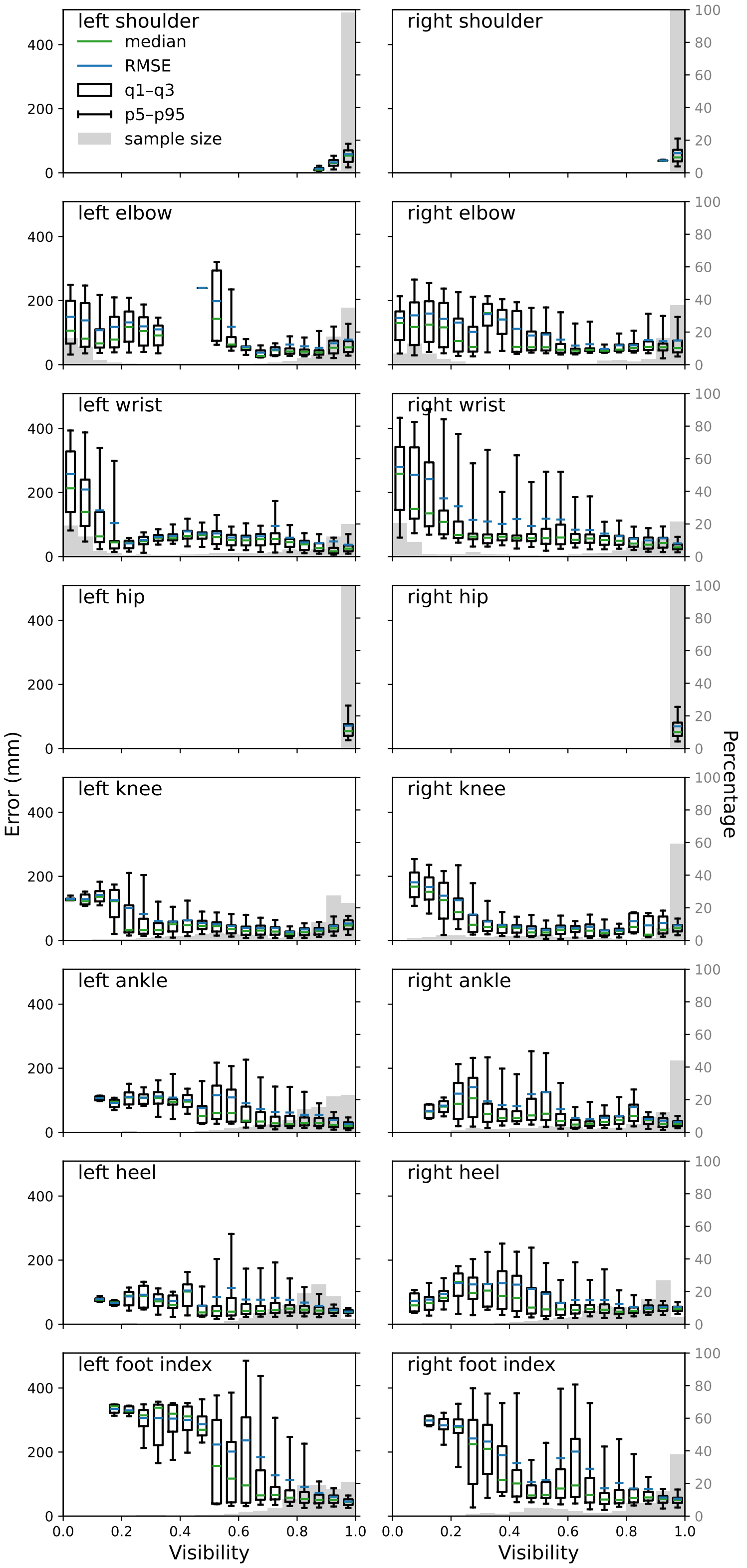

Figure S3. Joint position error distributions across visibility bins for individual joints.

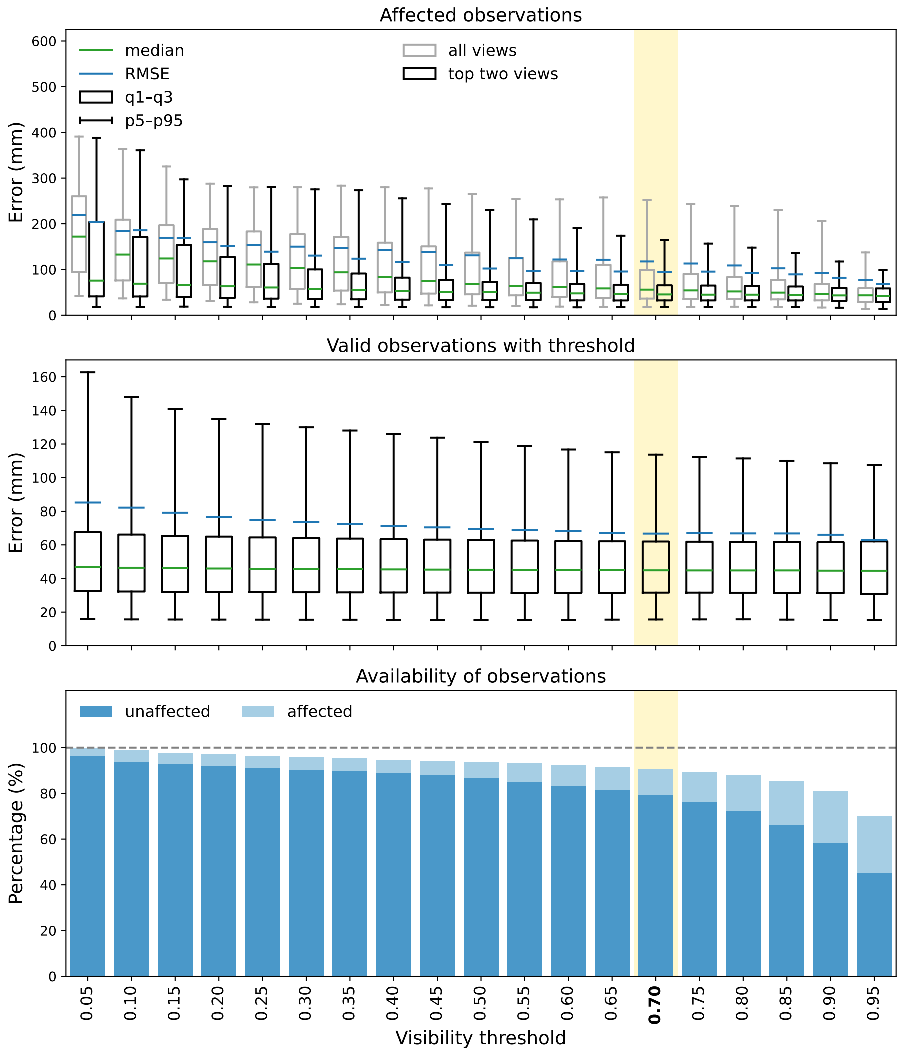

Figure S4. Sensitivity of joint position error and data availability to the visibility threshold. For each joint observation, camera-view visibility scores are ordered as $v_1 \geq v_2 \geq v_3$. At threshold t, observations are unaffected ($v_3 \geq t$; all three views used), affected ($v_3 < t \leq v_2$; lowest-visibility view excluded), or missing ($v_2 < t$; fewer than two views available). Data are pooled across trials and joints, excluding the chest and pelvis. Top: Errors for affected observations reconstructed using three views (gray) or the two highest-visibility views (black), evaluated on identical observations at each threshold. Middle: Errors for all valid observations after filtering; the retained observations change with the threshold. Bottom: Percentages of unaffected (dark blue) and affected (light blue) observations relative to all observations with marker-based data. The gap to the dashed 100% line represents missing observations. The selected threshold of 0.7 is highlighted.

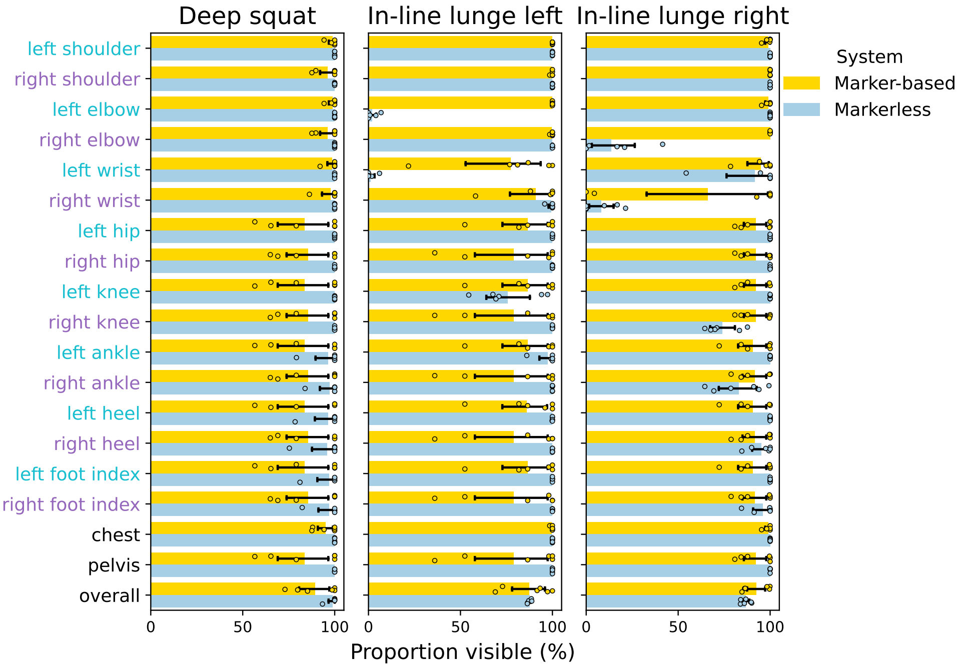

(**a**)

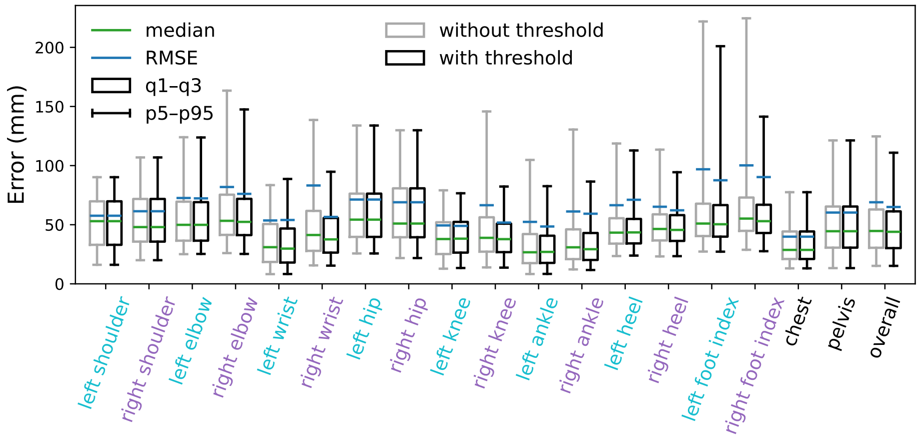

(**b**)

Figure S5. (**a**) Data availability in each trial for every joint and exercise: marker-based observations present (yellow) and markerless observations reconstructed with visibility ≥ 0.7 (blue). (**b**) Joint position error with and without visibility filtering on identical valid observations ($v_2 \geq t$), pooled across trials. Camera-view scores are ordered as $v_1 \geq v_2 \geq v_3$, and $t = 0.7$. Both affected ($v_3 < t \leq v_2$) and unaffected ($v_3 \geq t$) observations are included.

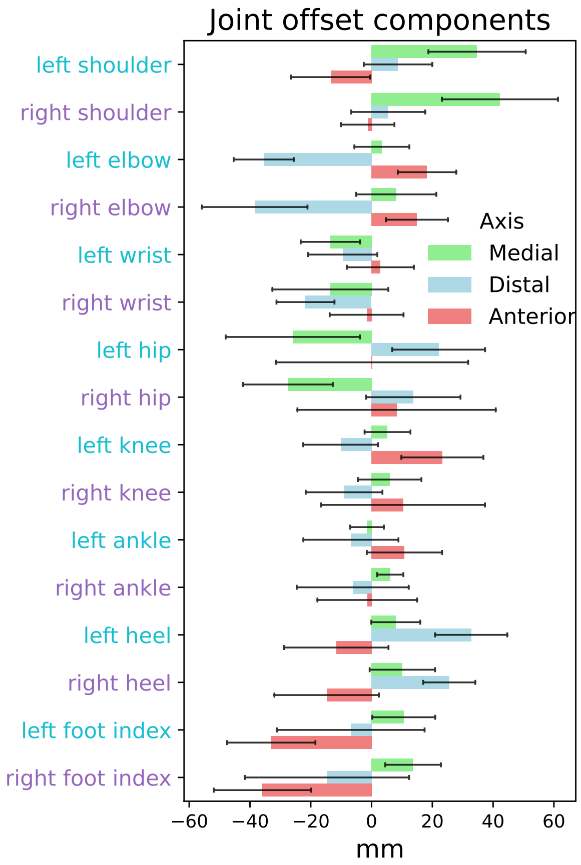

Figure S6. Components of the mean joint offset at the original scale along the anatomical axes. Error bars represent the standard deviation across subjects.

Table S4. Joint offset components along the anatomical axes (mm), normalized to a reference height of 1.70 m. Values are reported as the mean (SD) across subjects. Positive components indicate medial, distal, and anterior directions; negative components indicate lateral, proximal, and posterior directions, respectively.

Joint	Medial	Distal	Anterior
Left shoulder	34.1 (13.5)	8.7 (10.5)	−13.3 (11.5)
Right shoulder	41.9 (16.6)	5.1 (11.4)	−1.3 (8.0)
Left elbow	3.4 (8.3)	−35.5 (9.0)	18.1 (8.7)
Right elbow	7.9 (11.9)	−38.1 (15.0)	14.9 (9.1)
Left wrist	−13.6 (9.0)	−9.4 (10.0)	2.9 (9.9)
Right wrist	−13.8 (17.3)	−22.0 (9.1)	−1.8 (10.9)
Left hip	−25.3 (19.8)	22.2 (13.7)	−0.4 (28.6)
Right hip	−27.4 (13.4)	13.6 (13.8)	7.7 (29.1)
Left knee	5.1 (6.7)	−10.1 (11.0)	23.3 (11.9)
Right knee	5.8 (9.8)	−8.8 (11.1)	9.8 (24.1)
Left ankle	−1.5 (5.1)	−7.0 (15.2)	11.2 (12.2)
Right ankle	6.2 (4.1)	−6.4 (17.5)	−1.3 (15.6)
Left heel	7.9 (7.0)	33.1 (12.1)	−11.5 (15.4)
Right heel	10.0 (10.1)	25.8 (8.5)	−14.8 (15.1)
Left foot index	10.5 (9.6)	−6.3 (22.3)	−32.8 (12.7)
Right foot index	13.5 (8.2)	−14.3 (24.6)	−35.9 (14.9)

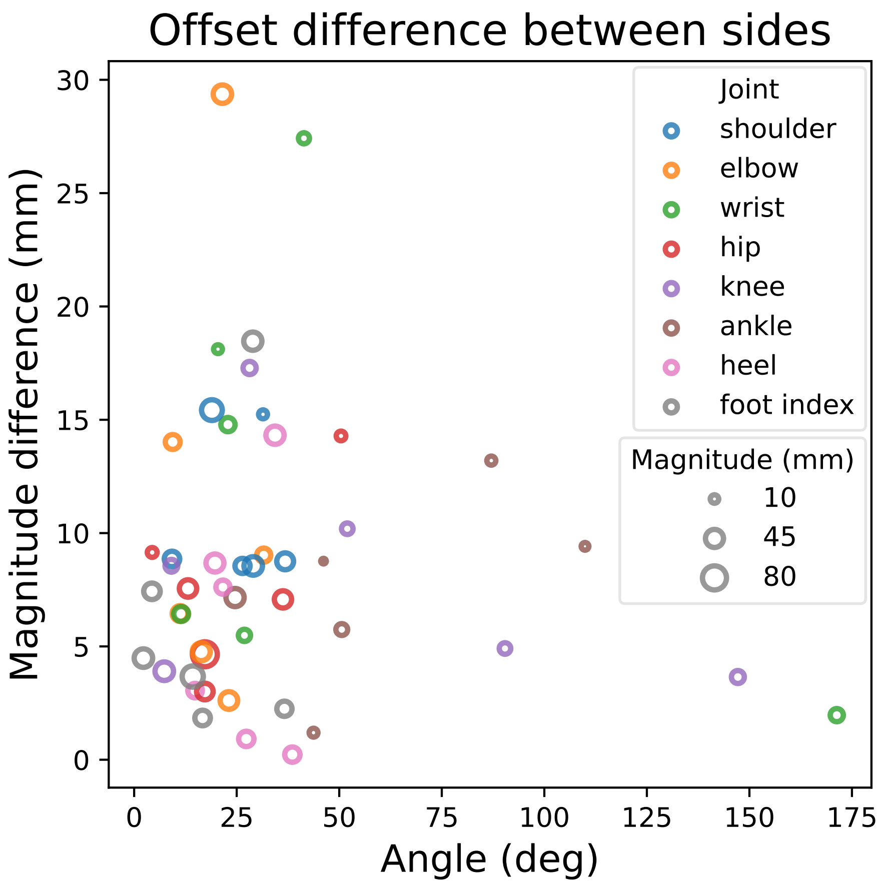

Figure S7. Differences in joint offset magnitude and direction between the left and right sides for all subjects. Marker size represents the mean offset magnitude across both sides.

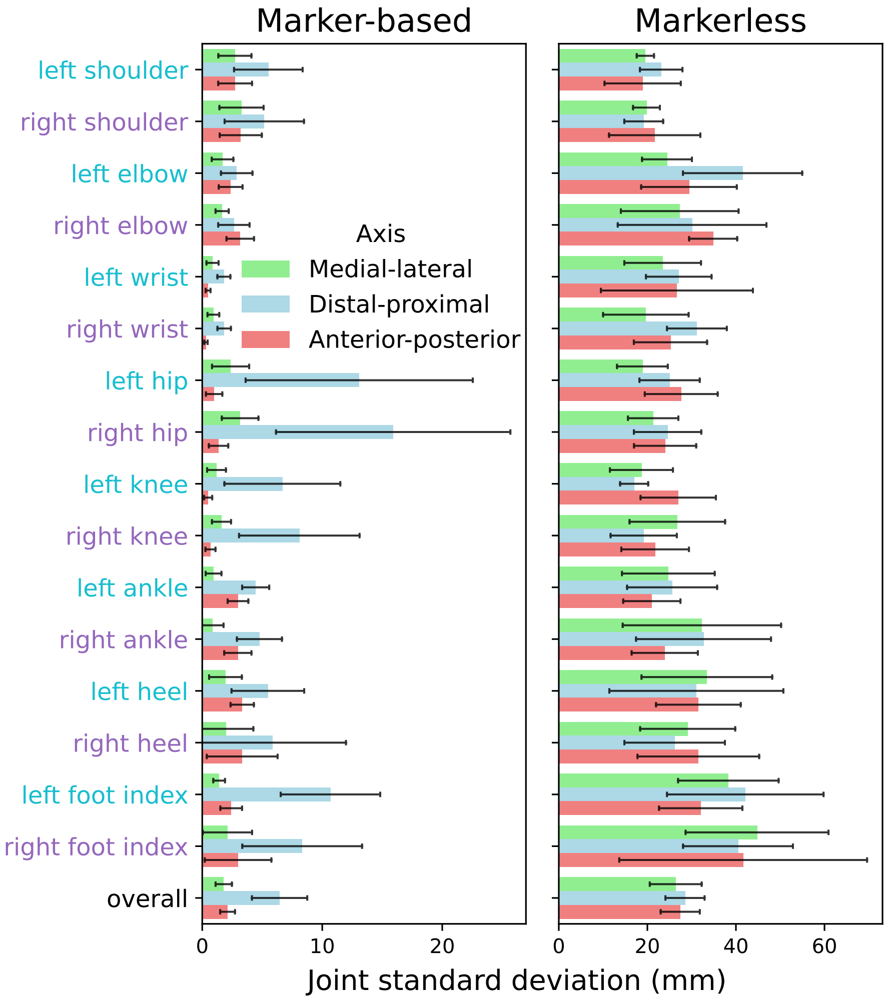

Figure S8. Residual variability of joint positions in the segment reference frame, expressed as the standard deviation along each anatomical axis. Error bars represent the standard deviation across subjects.

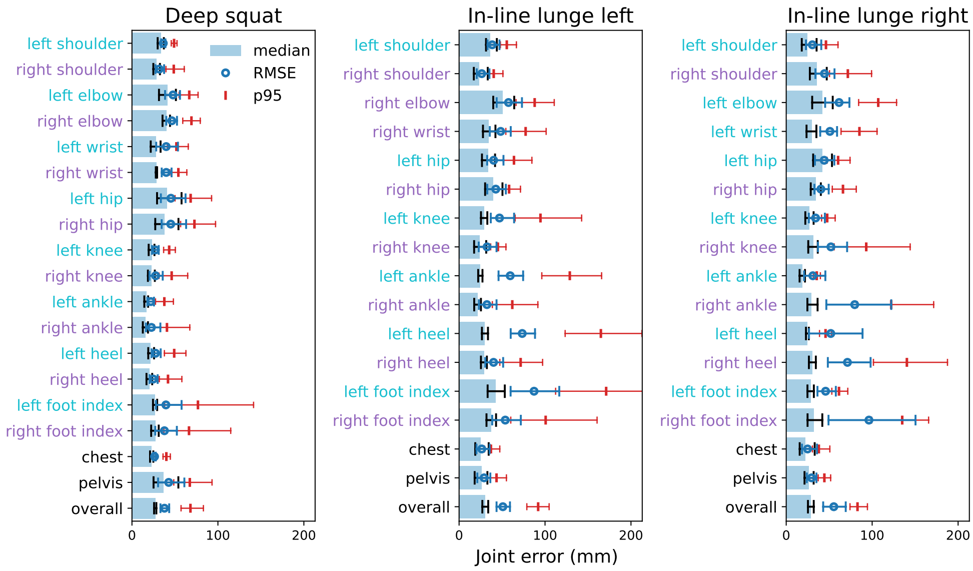

Figure S9. Joint position error statistics after offset correction for each exercise. Error bars show 95% bootstrap confidence intervals across six participants.

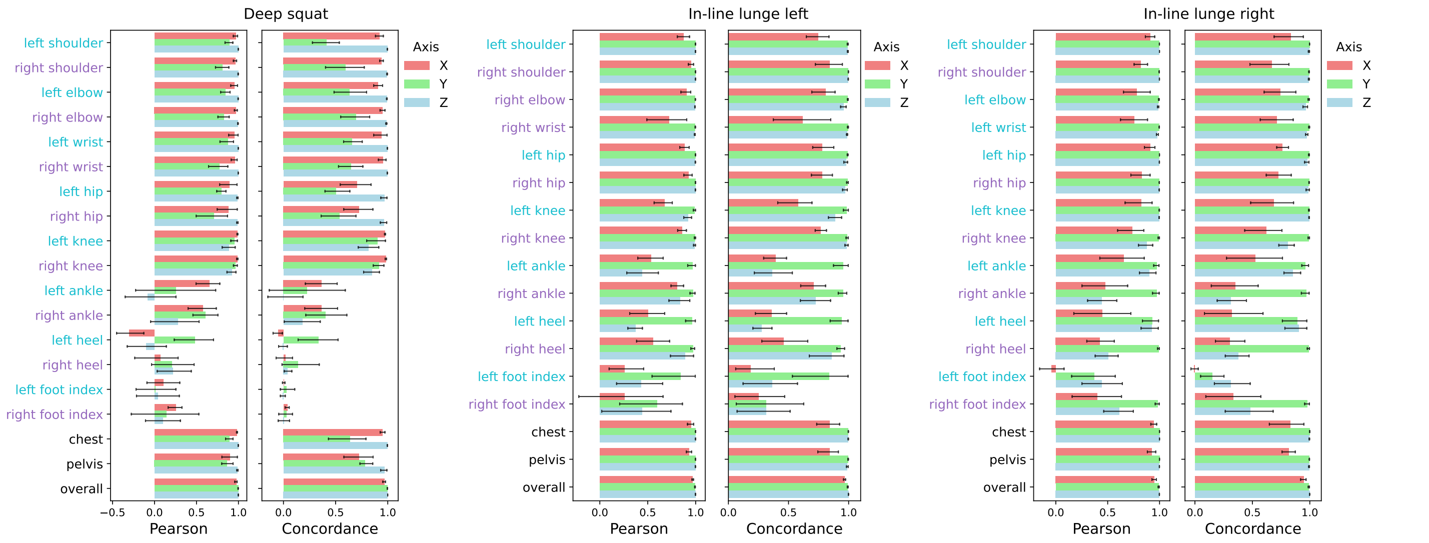

(**a**) (**b**) (**c**)

Figure S10. Pearson correlation coefficients and Lin's concordance correlation coefficients for offset-corrected joint positions along each world axis. (**a**) Deep squat. (**b**) In-line lunge (left). (**c**) In-line lunge (right). Error bars show 95% bootstrap confidence intervals across six participants.

Table S5. Overall joint position correlations along each world axis, pooling all joints within each trial. Pearson correlation coefficients (PCCs) and Lin's concordance correlation coefficients (CCCs) are reported as the mean (SD) across trials. "Original" denotes uncorrected estimates; "Offset" denotes offset-corrected estimates.

		X	Y	Z
**PCC**	Original	0.9549 (0.0229)	0.9902 (0.0069)	0.9981 (0.0009)
	Offset	0.9646 (0.0224)	0.9944 (0.0053)	0.9987 (0.0007)
**CCC**	Original	0.9445 (0.0280)	0.9884 (0.0077)	0.9977 (0.0010)
	Offset	0.9604 (0.0231)	0.9940 (0.0053)	0.9987 (0.0007)

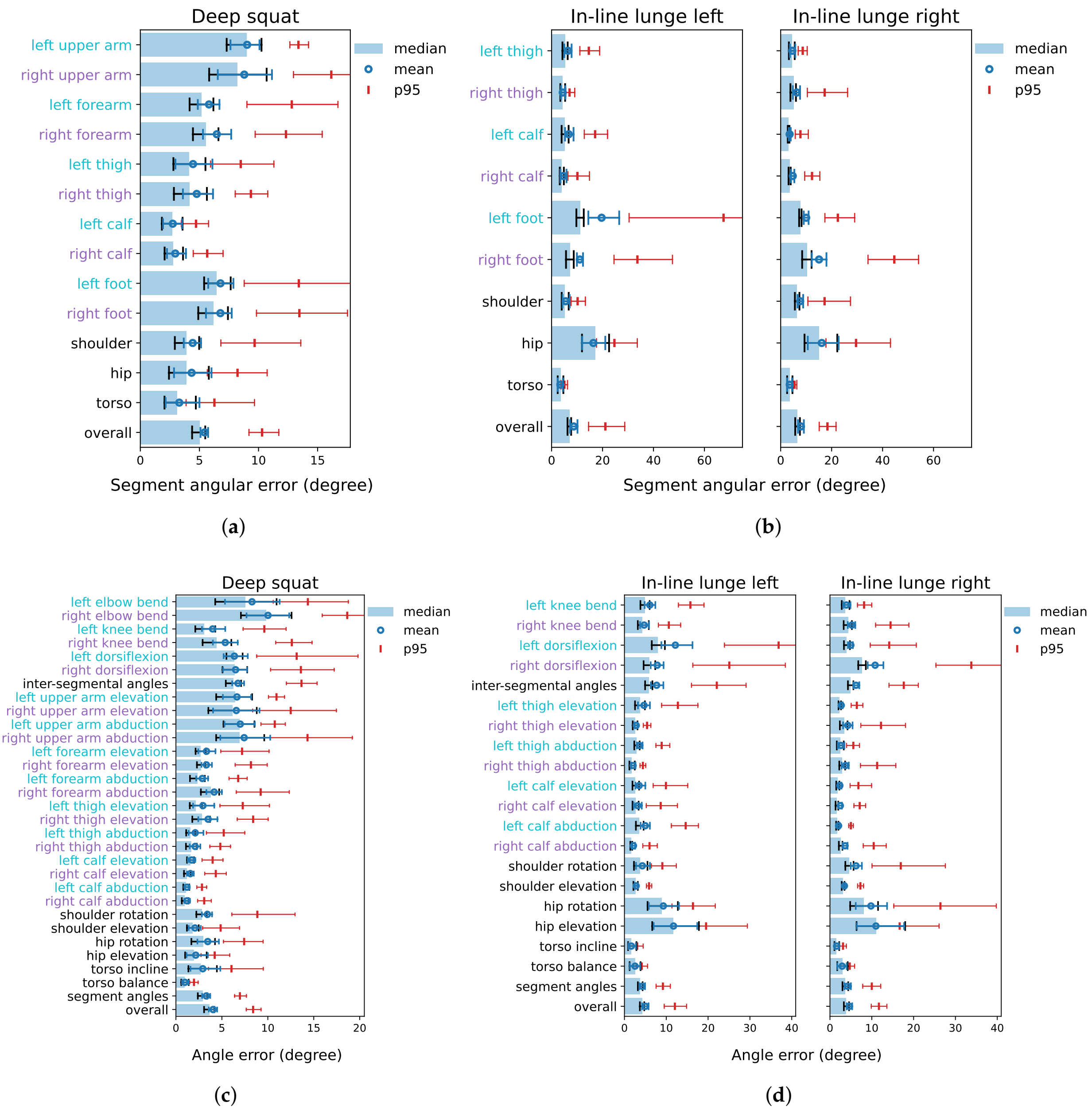

Figure S11. Segment orientation error statistics after offset correction for each exercise: (**a**) deep squat and (**b**) in-line lunge. Error statistics for inter-segmental and segment angles after offset correction: (**c**) deep squat and (**d**) in-line lunge. All error bars show 95% bootstrap confidence intervals across six participants.

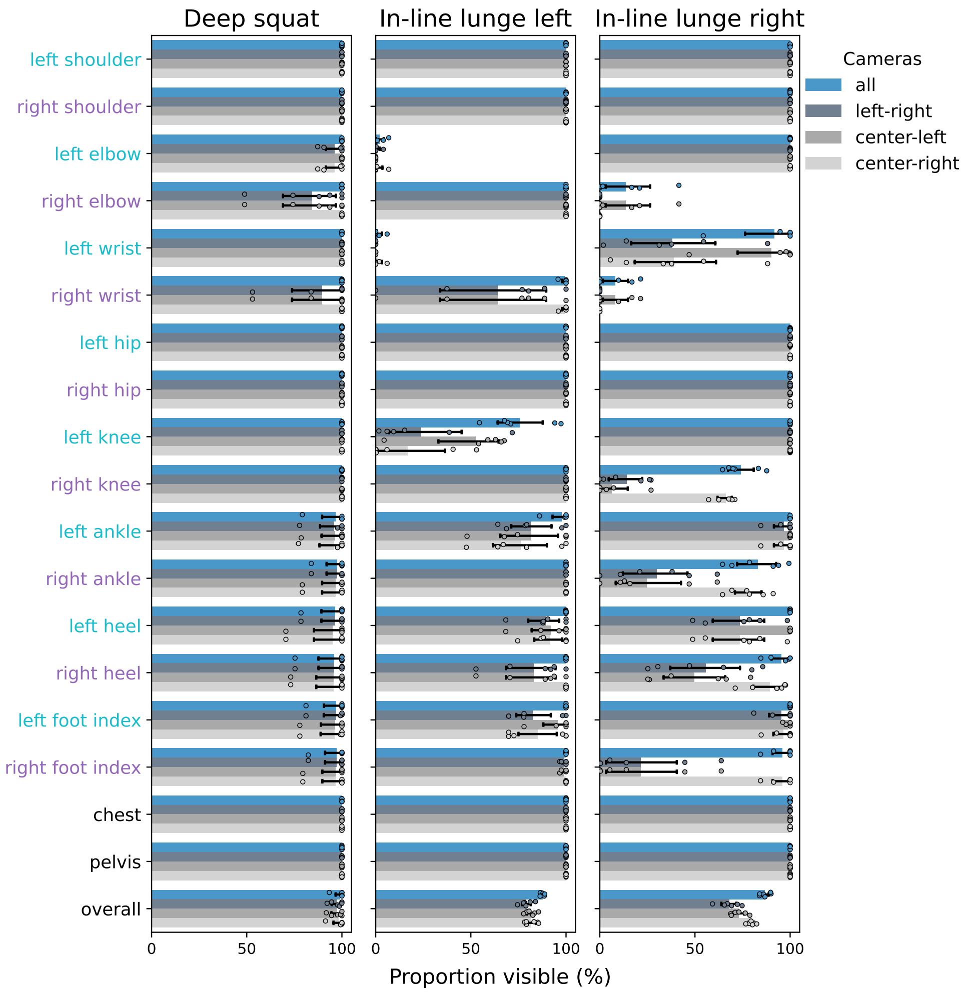

(**a**)

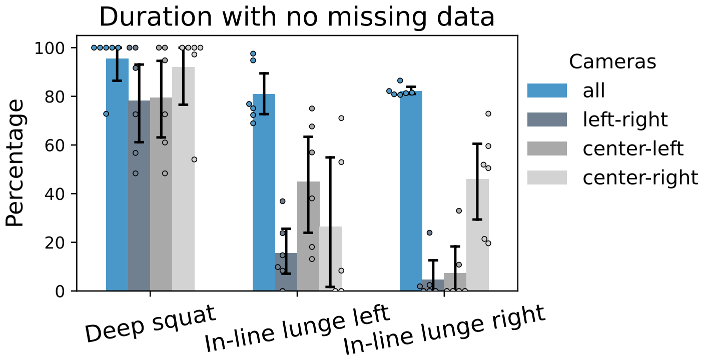

(**b**)

Figure S12. Markerless data availability for each exercise under different camera configurations. “All” (blue) denotes three cameras; the other labels (gray) denote camera pairs. (**a**) Percentage of valid observations for each joint (visibility ≥ 0.7). (**b**) Percentage of each trial in which all joints required for the metrics were simultaneously available. Error bars show 95% bootstrap confidence intervals across six participants.

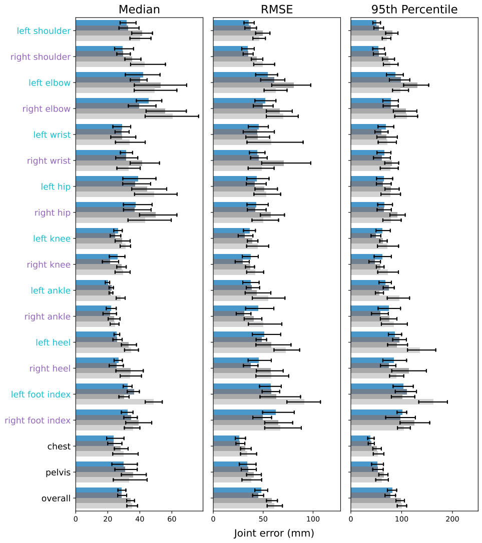

(**a**)

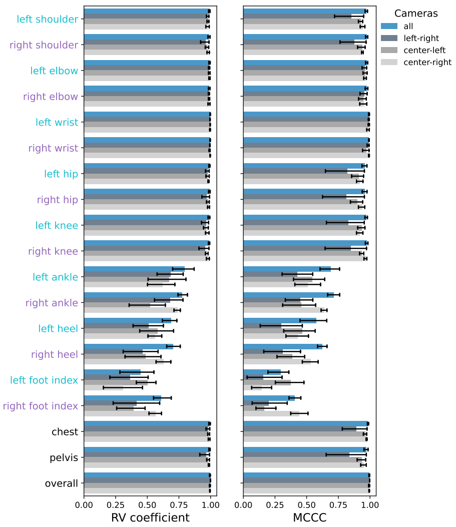

(**b**)

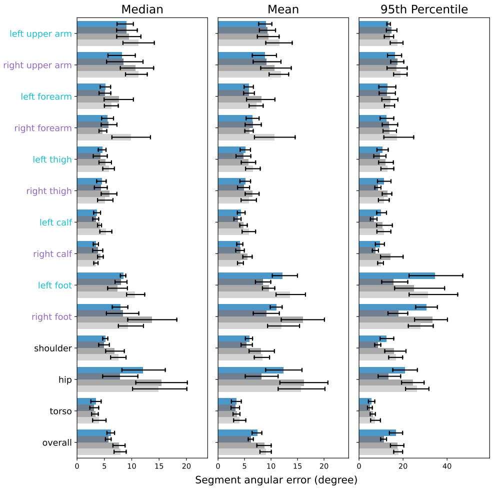

(**c**)

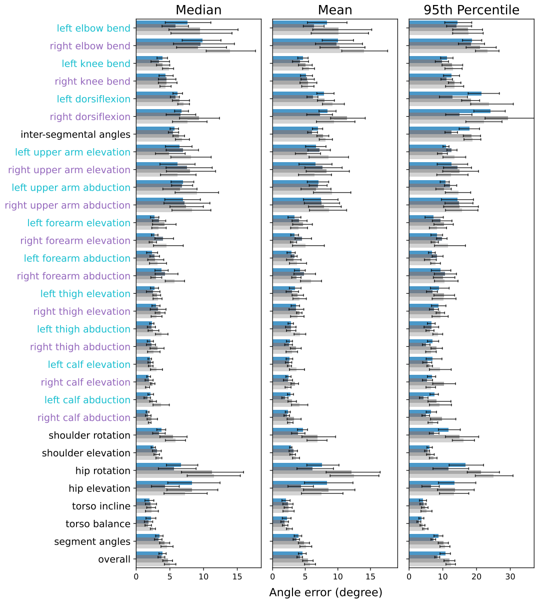

(**d**)

Figure S13. Offset-corrected markerless-system performance on each camera configuration's valid observations. "All" (blue) denotes three cameras; the other labels (gray) denote camera pairs. (**a**) Joint position error statistics. (**b**) Multivariate correlations for joint positions: RV-coefficient (left) and matrix-based concordance correlation coefficient (MCCC; right). (**c**) Segment orientation error statistics. (**d**) Error statistics for inter-segmental and segment angles. Bars show means and 95% bootstrap confidence intervals across six participants (Section S1).

Table S6. Overall offset-corrected joint position error statistics (mm) on each camera configuration's valid observations, pooling all joints within each trial. Values are reported as the mean (SD) across trials.

	**Median**	**RMSE**	**95th percentile**
All	28.6 (3.7)	48.0 (14.2)	81.0 (18.4)
Left–right	28.7 (4.4)	44.8 (9.7)	77.2 (17.6)
Center–left	34.3 (4.5)	58.5 (15.5)	97.7 (19.8)
Center–right	35.2 (5.2)	61.1 (18.9)	100.3 (23.7)

Table S7. Comparison of selected multi-camera markerless motion capture studies. (**a**) System configurations and validation protocols.

Study, sample, and tasks	RGB camera configuration	Estimator and reconstruction	Reference and synchronization	Processing and throughput
**Ceriola et al. (2024)** [2]<br>10 healthy men; treadmill walking and running.	Two converging views; 1920 × 1080, 60 Hz; separation NR.	BlazePose Heavy and other estimators, including RTMPose-L; calibrated DLT; segment angles.	Six-camera Vicon, 120 Hz; Plug-in Gait hip and knee angles. RGB synchronization via OBS; reference alignment by knee cross-correlation.	Recorded-video analysis; measured throughput NR.
**Dill et al. (2024)** [3]<br>Nine analyzed participants; 81 squat recordings.	Two smartphones, approximately 90°; 1080 × 1920, 30 Hz.	MediaPipe Pose; selected epipolar DLT uses calibrated intrinsics and pose-derived extrinsics.	Eleven-camera Qualisys, 100 Hz; 12 common joint positions and geometric angles. Temporal synchronization NR.	Offline analysis; measured throughput NR.
**Samani et al. (2026)** [4]<br>10 healthy men; eight static poses and overhead lifting.	Six camera pairs evaluated separately, 15 Hz; selected pairs approximately 45°/135°.	MediaPipe Pose (model complexity 1); checkerboard-calibrated triangulation; nine geometric angles.	Qualisys; reference downsampled to 15 Hz. Synchronization procedure NR.	Processing backend and measured throughput NR.
**Uhlrich et al. (2023), OpenCap** [5]<br>10 healthy adults; walking, squats, sit-to-stand, and drop jumps.	Primary configuration: two smartphones, 90°; 720 × 1280, 60 Hz.	HRNet/OpenPose; calibrated, confidence-weighted DLT; anatomical-marker augmentation and OpenSim inverse kinematics.	Eight-camera optical system, 100 Hz; anatomical markers and model-based joint rotations. Inter-video cross-correlation and reference time alignment.	Cloud processing of recorded trials; live throughput NR.
**Bajrami et al. (2025)** [6]<br>Sample size NR; four right-arm gestures, five repetitions each.	Two or three RGB views; 640 × 480, 30 Hz; reported angular differences approximately 70°/75°.	MediaPipe Pose; calibrated DLT, monocular 3D, and fusion alternatives. Selected errors use three-view DLT.	Twelve-camera OptiTrack, 120 Hz; shoulder, elbow, and wrist positions. Host-clock synchronization and nearest-timestamp matching.	Offline image inference; measured throughput NR.
**Nakano et al. (2020)** [7]<br>Two healthy men; walking, jumping, and throwing.	Five views; 1920 × 1080 at 120 Hz (1K), or 3840 × 2160 at 30 Hz (4K).	OpenPose BODY_25; calibration-pole DLT and zero-lag filtering.	Sixteen-camera optical system, 200 Hz; marker-derived joint centers. Manual temporal alignment using a light event.	Offline GPU processing; measured live throughput NR.
**Present study**<br>Six healthy adults; deep squat and in-line lunge.	Three views; 1024 × 768, 30 Hz; adjacent/outer pairs 37.5°/75°.	MediaPipe Pose (model complexity 1); checkerboard-calibrated, visibility-filtered DLT; landmark positions and geometric angles.	Eight-camera Vicon, 100 Hz; Plug-in Gait and heel/foot-index markers. RGB host timestamps; reference alignment by trajectory cross-correlation.	Local CPU processing at approximately 20 FPS; feedback latency unmeasured.

NR denotes a detail not sufficiently reported in the reviewed source. Camera acquisition rate and processing throughput are distinct. Samani evaluated camera pairs rather than reconstructing from eight views; Bajrami used only the RGB streams of RGB-D cameras. Residual synchronization uncertainty was not independently quantified in these comparisons.

Table S7. Comparison of selected multi-camera markerless motion capture studies, continued. (**b**) Selected positional and angular errors.

Study and configuration	Position error (mm)	Angular error (°)	Outcome definition and evaluation notes
**Ceriola** [2] running	NR.	Hip/knee waveform RMSE: MediaPipe 3.5 (1.5)/4.7 (1.7); RTMPose-L/Halpe 3.4 (1.6)/4.9 (2.6).	Sagittal waveform RMSE after removing each gait cycle's mean angle offset; mean (SD). Both models use the same protocol.
**Dill** [3] epipolar, unfiltered	Median recording-level position RMSE after fitting: 30.9.	Median recording-level knee RMSE: left 10.3, right 7.7.	81 recordings; 12 common joint positions fitted to reference before error calculation. Fitting transformation and scope NR; geometric knee angles.
**Samani** [4] selected pairs	NR.	Nine-angle MAE, static/dynamic: pair 1–3, 9.28/12.92; pair 3–7, 25.33/30.59. Pair 1–3 knee MAE, L/R: static 3.13/3.23; dynamic 6.20/6.81.	Approximately 45°/135° pairs. MAE averaged over nine angles; knee-specific values shown separately. Results depend on configuration and task.
**OpenCap** [5] two cameras	Mean Euclidean anatomical-marker error: HRNet 32; OpenPose high-accuracy/default 31/35.	Mean rotational MAE: HRNet 4.5; OpenPose high-accuracy/default 4.3/4.7.	17 augmented anatomical markers; 18 rotational degrees of freedom, pooled across activities. Marker error uses optimized time shift and mean-position alignment; biomechanical constraints applied.
**Bajrami** [6] three-view DLT with moving average	Reported mean joint RMSE: drinking 96.1; eating 106.8; zipper 52.9; nose 77.1.	NR.	Mean across three right-arm joint RMSEs, averaged over five repetitions. Smoothing tuned to reference by task/configuration; aggregation from per-axis to joint RMSE incompletely specified.
**Nakano** [7] five views	Coordinate-wise MAE range: 1K 4.32–47.3; 4K 4.09–66.8.	NR.	Source Table 1 ranges across task/joint/axis MAEs rather than Euclidean distances. Six right-side joints; retrospective left/right swap correction and zero-lag filtering.
**Present study** uncorrected	Mean trial-level median distance: 45.4 (7.6); mean trial-level distance RMSE: 64.2 (11.0).	Mean overall angle error: 6.4 (0.9); mean segment orientation error: 9.6 (1.5).	Mean (sample SD) of six participant summaries. Joint distances pooled within trials; angular errors averaged across metrics. Board-derived global mapping; no offset correction.
**Present study** across-subject correction	Mean trial-level median distance: 38.1 (7.6); mean trial-level distance RMSE: 56.4 (10.7).	Mean overall angle error: 5.2 (0.9); mean segment orientation error: 8.5 (1.6).	Mean (sample SD) of six participant summaries. Offsets from other subjects applied in marker-based segment frames; excludes 4.1% of observations lacking frames.

Study-specific outputs and corrections prevent a cross-study performance ranking. MAE, RMSE, and median distance are not converted; parentheses denote SD. Geometric angles, model-based joint rotations, and segment orientation errors are distinct. Present-study in-sample and within-subject corrections: manuscript Table 2.

S1. Participant-level variability and uncertainty

Methods

For each correction scheme or camera configuration, trial-level statistics were averaged within each participant, giving equal weight to the deep squat and two in-line lunge trials. The primary outcomes were trial-level median joint position errors and trial-level mean segment orientation and angle metric errors. For the wrist and elbow joints and their associated metrics, which were excluded from in-line lunge evaluations, participant summaries used the deep squat trial only. We then calculated the mean and sample SD across six participants. This averaging procedure was applied to each statistic shown in Figures 5 and S13. For the exercise-specific summaries in Figures S9–S12, each participant contributed one trial per exercise and performed-side condition. Table S8 reports uncorrected results and the three offset-correction schemes in manuscript Table 2; the exercise-specific comparisons in Figure S14 use uncorrected results only.

For Figures 5, S13, and S14 and Table S8, exploratory 95% percentile bootstrap intervals were calculated from 10,000 resamples of six participant summaries with replacement. For corrected results, offsets were not re-estimated in each bootstrap sample, so the intervals do not include uncertainty in offset estimation. The analyses were descriptive: six independent participants provide limited precision for population comparisons and little information for checking distributional assumptions. We therefore did not perform confirmatory hypothesis tests or treat repeated frames and trials as additional independent participants.

Results

Uncorrected participant means of trial-level median joint position error ranged from 37.3 to 57.0 mm (Table S8). Deep squat, left in-line lunge, and right in-line lunge cohort means were 43.3, 47.7, and 45.2 mm for joint position error; 7.5, 11.2, and 10.2° for segment orientation error; and 6.1, 6.7, and 6.3° for overall angle error (Figure S14). These differences combine task, visibility, and participant effects. Left and right identify the performed lunge condition, not the anatomical side of evaluated joints. Each session contained different participants, so session effects cannot be separated from participant differences.

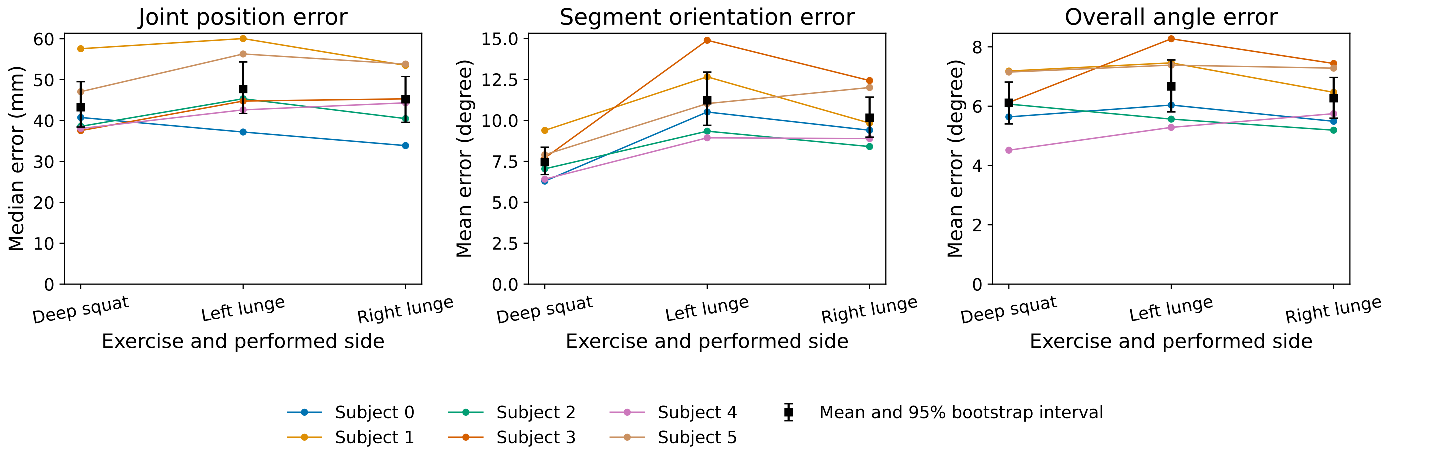

Figure S14. Uncorrected joint position, segment orientation, and overall angle errors across participants and exercise conditions. Colored points and lines identify participants; black squares and error bars show cohort means and 95% participant-bootstrap intervals. Joint position errors are trial-level medians; segment orientation and overall angle errors are trial-level means.

Table S8. Joint position, segment orientation, and overall angle errors for six participants, using equal trial weights within participants. Joint position errors average trial-level medians; segment orientation and overall angle errors average trial-level means. The final columns give the mean (sample SD) and 95% participant-bootstrap interval. Correction schemes follow manuscript Table 2.

Correction	Subject 0	Subject 1	Subject 2	Subject 3	Subject 4	Subject 5	Mean (SD)	95% interval
**Joint position error (mm)**								
Uncorrected	37.3	57.0	41.5	42.5	41.7	52.4	45.4 (7.6)	[40.3, 51.3]
In-sample	25.9	34.0	29.6	25.3	25.9	30.9	28.6 (3.5)	[26.3, 31.3]
Within-subject	26.1	34.4	29.9	25.9	26.1	31.7	29.0 (3.6)	[26.7, 31.8]
Across-subject	37.9	52.5	35.2	32.3	32.2	38.3	38.1 (7.6)	[33.7, 44.3]
**Segment orientation error (°)**								
Uncorrected	8.7	10.6	8.3	11.7	8.1	10.3	9.6 (1.5)	[8.6, 10.7]
In-sample	7.5	8.8	6.1	8.4	5.7	7.7	7.4 (1.2)	[6.4, 8.3]
Within-subject	7.5	8.8	6.2	8.6	5.7	7.8	7.4 (1.3)	[6.5, 8.4]
Across-subject	9.7	9.9	6.7	10.0	6.3	8.6	8.5 (1.6)	[7.3, 9.7]
**Overall angle error (°)**								
Uncorrected	5.7	7.0	5.6	7.3	5.2	7.3	6.4 (0.9)	[5.7, 7.0]
In-sample	4.7	5.6	3.8	5.0	3.4	4.9	4.6 (0.8)	[4.0, 5.2]
Within-subject	4.7	5.6	3.8	5.1	3.4	5.0	4.6 (0.8)	[4.0, 5.2]
Across-subject	5.6	6.1	4.3	6.0	3.8	5.3	5.2 (0.9)	[4.5, 5.8]

S2. Sensitivity to inter-system alignment

Methods

Using uncorrected results only, we compared quadratic and linear interpolation for estimating the inter-system time lag, with other alignment settings unchanged. For each interpolation method, the estimated lag was evaluated without an additional shift and at plus or minus one markerless frame, defined by the recording's median frame interval. The 100-Hz interpolation grid was used only for lag estimation and did not add temporal information. Final marker-based trajectory resampling remained quadratic, and errors were evaluated at the native markerless timestamps.

For each outcome, the same valid observations were used across all six conditions, and the quadratic zero-shift baseline was recomputed on this common set. Paired changes from this baseline were averaged within participants and summarized using the bootstrap procedure in Section S1.

Results and interpretation

The recording-specific frame intervals ranged from 32.63 to 36.18 ms. Quadratic and linear lag estimates agreed in 15 of 18 recordings; linear estimates were 10 ms lower in three. The quadratic zero-shift baseline, evaluated on matched observations, gave a mean trial-level median joint position error of 45.42 mm and mean segment orientation and overall angle errors of 9.60° and 6.34°, respectively. At zero shift, linear lag estimation increased these means by 0.071 mm, 0.008°, and 0.007°. Across all six conditions, including one-frame shifts, the largest mean increases were 0.853 mm, 0.156°, and 0.133° (Figure S15).

Changes in cohort means do not bound trial or observation errors. Lag estimation used some evaluated joints, so agreement may be optimistic; this local check does not remove selection bias or test broader alignment-interval choices. Inter-camera jitter could not be quantified because individual-camera timestamps were not retained.

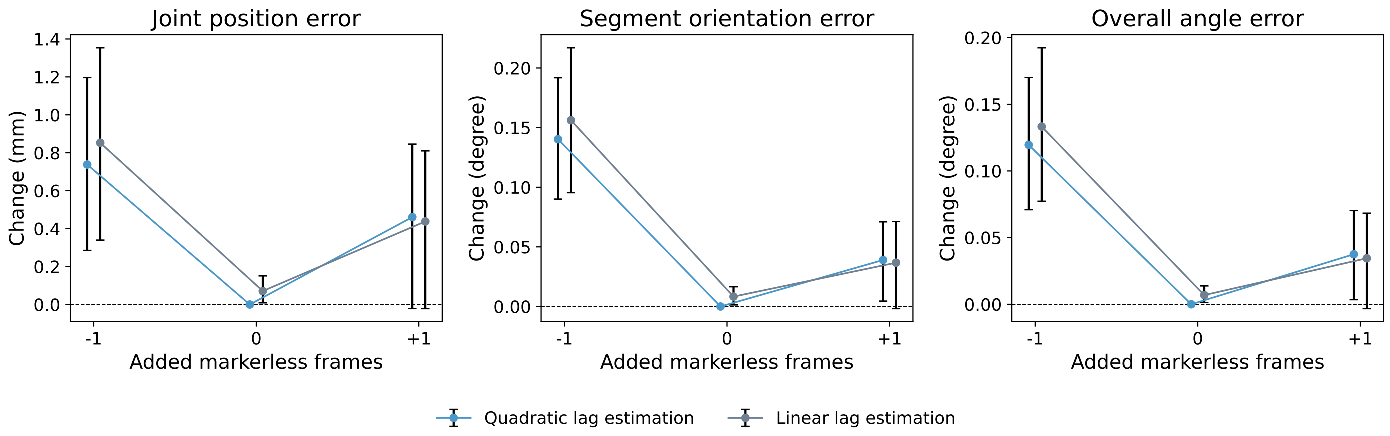

Figure S15. Mean paired changes in uncorrected errors from the quadratic zero-shift baseline, using identical observations across all six conditions. Error bars show 95% participant-bootstrap intervals; the dashed line marks no change. One frame is the recording-specific median markerless interval (32.63–36.18 ms).

References

1. Gray Cook, Lee Burton, Barbara J Hoogenboom, and Michael Voight. Functional movement screening: the use of fundamental movements as an assessment of function-part 1. *International Journal of Sports Physical Therapy*, 9(3):396, 2014.
2. Luca Ceriola, Juri Taborri, Marco Donati, Stefano Rossi, Fabrizio Patanè, and Ilaria Mileti. Comparative analysis of markerless motion capture systems for measuring human kinematics. *IEEE Sensors Journal*, 24(17):28135–28144, 2024.
3. Sebastian Dill, Arjang Ahmadi, Martin Grimmer, Dennis Haufe, Maurice Rohr, Yanhua Zhao, Maziar Sharbafi, and Christoph Hoog Antink. Accuracy evaluation of 3D pose reconstruction algorithms through stereo camera information fusion for physical exercises with MediaPipe Pose. *Sensors*, 24(23):7772, 2024.
4. Ali Sahraneshin Samani, Mohsen Razeghi, Mohammad Abdoli-Eramaki, and Alireza Choobineh. Optimizing camera placement in multi-view markerless motion capture: a validation study against gold-standard kinematics for static and dynamic tasks. *BMC Research Notes*, 19:303, 2026. https://doi.org/10.1186/s13104-026-07886-4.
5. Scott D. Uhlrich, Antoine Falisse, Łukasz Kidziński, Julie Muccini, Michael Ko, Akshay S. Chaudhari, Jennifer L. Hicks, and Scott L. Delp. OpenCap: Human movement dynamics from smartphone videos. *PLOS Computational Biology*, 19(10):e1011462, 2023. https://doi.org/10.1371/journal.pcbi.1011462.
6. Albin Bajrami, Gloria Beraldo, Matteo Claudio Palpacelli, Tapio Heikkila, and Gabriella Cortelessa. Markerless upper limb motion tracking: A comparative evaluation of multi-view approaches. In *2025 IEEE International Conference on Metrology for eXtended Reality, Artificial Intelligence and Neural Engineering (MetroXRAINE)*, pages 818–823. IEEE, 2025.
7. Nobuyasu Nakano, Tetsuro Sakura, Kazuhiro Ueda, Leon Omura, Arata Kimura, Yoichi Iino, Senshi Fukashiro, and Shinsuke Yoshioka. Evaluation of 3D markerless motion capture accuracy using openpose with multiple video cameras. *Frontiers in Sports and Active Living*, 2:50, 2020.
